# A mechanistic framework for *a priori* pharmacokinetic predictions of orally inhaled drugs

**DOI:** 10.1371/journal.pcbi.1008466

**Published:** 2020-12-15

**Authors:** Niklas Hartung, Jens Markus Borghardt

**Affiliations:** 1 Institute of Mathematics, University of Potsdam, Potsdam, Germany; 2 Drug Discovery Sciences, Research DMPK, Boehringer Ingelheim Pharma GmbH & Co. KG, Biberach, Germany; University at Buffalo - The State University of New York, UNITED STATES

## Abstract

The fate of orally inhaled drugs is determined by pulmonary pharmacokinetic processes such as particle deposition, pulmonary drug dissolution, and mucociliary clearance. Even though each single process has been systematically investigated, a quantitative understanding on the interaction of processes remains limited and therefore identifying optimal drug and formulation characteristics for orally inhaled drugs is still challenging. To investigate this complex interplay, the pulmonary processes can be integrated into mathematical models. However, existing modeling attempts considerably simplify these processes or are not systematically evaluated against (clinical) data. In this work, we developed a mathematical framework based on physiologically-structured population equations to integrate all relevant pulmonary processes mechanistically. A tailored numerical resolution strategy was chosen and the mechanistic model was evaluated systematically against data from different clinical studies. Without adapting the mechanistic model or estimating kinetic parameters based on individual study data, the developed model was able to predict simultaneously (i) lung retention profiles of inhaled insoluble particles, (ii) particle size-dependent pharmacokinetics of inhaled monodisperse particles, (iii) pharmacokinetic differences between inhaled fluticasone propionate and budesonide, as well as (iv) pharmacokinetic differences between healthy volunteers and asthmatic patients. Finally, to identify the most impactful optimization criteria for orally inhaled drugs, the developed mechanistic model was applied to investigate the impact of input parameters on both the pulmonary and systemic exposure. Interestingly, the solubility of the inhaled drug did not have any relevant impact on the local and systemic pharmacokinetics. Instead, the pulmonary dissolution rate, the particle size, the tissue affinity, and the systemic clearance were the most impactful potential optimization parameters. In the future, the developed prediction framework should be considered a powerful tool for identifying optimal drug and formulation characteristics.

## Author summary

The use of orally inhaled drugs for treating lung diseases is appealing since they have the potential for lung selectivity, i.e. high exposure at the site of action –the lung– without excessive side effects. However, the degree of lung selectivity depends on a large number of factors, including physiochemical properties of drug molecules, patient disease state, and inhalation devices. To predict the impact of these factors on drug exposure and thereby to understand the characteristics of an optimal drug for inhalation, we develop a predictive mathematical framework (a “pharmacokinetic model”). In contrast to previous approaches, our model allows combining knowledge from different sources appropriately and its predictions were able to adequately predict different sets of clinical data. Finally, we compare the impact of different factors and find that the most important factors are the size of the inhaled particles, the affinity of the drug to the lung tissue, as well as the rate of drug dissolution in the lung. In contrast to the common belief, the solubility of a drug in the lining fluids is not found to be relevant. These findings are important to understand how inhaled drugs should be designed to achieve best treatment results in patients.

## Introduction

Oral drug inhalation can result in high pulmonary drug exposure while maintaining low systemic exposure. Compared to other routes of administration, this can provide higher local pulmonary efficacy, while simultaneously reducing systemic adverse effects (“lung selectivity”) [1–3]. Therefore, orally inhaled drugs are considered first-line therapy (amongst other treatment options) to treat respiratory diseases such as asthma bronchial or chronic obstructive pulmonary disease [4, 5].

While qualitatively, the pharmacodynamic (PD) selectivity for the lung was previously investigated, a sound quantitative understanding about the pulmonary pharmacokinetics (PK) is still lacking. Specific pulmonary PK processes after oral drug inhalation were studied in detail, such as the pulmonary particle deposition [6–8] or mucociliary clearance [9, 10]. For example, it is well understood that the central airway deposition increases with an increasing aerodynamic particle size [7] and that the mucociliary clearance depends on the localization in the airways [10]. Hence, the impact of mucociliary clearance strongly depends on particle deposition patterns. However, in contrast to investigations related to the individual processes, the interplay of the many pulmonary PK processes has received less attention. A comprehensive quantitative understanding of how these processes contribute to pulmonary and systemic PK, and therefore to lung selectivity after drug inhalation, is often still lacking [11–14]. Thus, identifying drug and formulation characteristics for orally inhaled drugs that maximize lung selectivity as well as long-lasting pulmonary efficacy is still challenging.

To gain a better understanding on the interplay of pulmonary PK processes, mechanistic modeling approaches can be applied. However, previous modeling approaches either reduced the given complexity or lack adequate model evaluation. For example, the mucociliary clearance was described as a first-order process [15, 16]. Other published population PK models did not differentiate between undissolved and dissolved drug and consider pulmonary drug absorption as a “one-way process”, i.e. back flow of drug to the lungs from the systemic disposition is not considered [17–19]. One mechanistic partial differential equation (PDE)-based model is available, which included all relevant pulmonary PK processes [20]. This model, however, was not evaluated against clinical data. Hence, to our knowledge no fully mechanistic model, with an adequate model evaluation based on clinical and *in vitro* data, is available. Consequently, there is currently no adequate framework to quantitatively identify the most impactful drug and formulation characteristics to achieve good lung selectivity.

**Fig 1:**
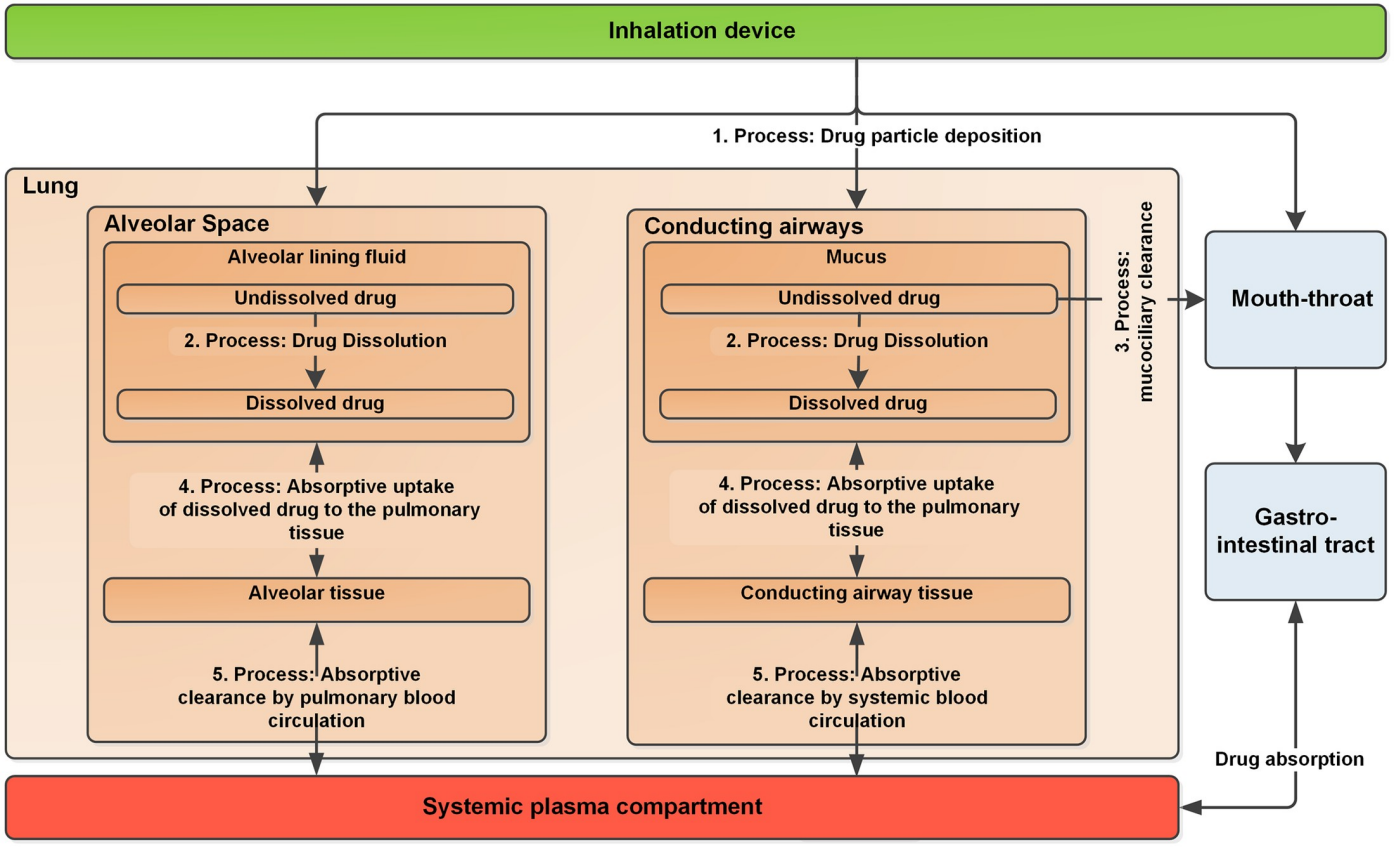
Overview of relevant pulmonary pharmacokinetic processes for orally inhaled drugs. Adapted and modified from [2].

In this work, we aimed at developing such a mechanistic pulmonary PK model to capture the complexity of all relevant pulmonary PK processes (compare Fig 1) and to determine which parameters are the most suitable optimization criteria to achieve optimal lung selectivity. The biggest mathematical challenge related to such a model is to adequately describe the joint effect of location-dependent nonlinear mucociliary clearance and particle size-dependent dissolution. To achieve this, a size- and location-structured PDE model was developed. The resulting PDE model was extensively evaluated, in particular based on clinical PK data for both budesonide and fluticasone propionate, as these inhaled drugs represent the clinically most studied compounds. Finally, a sensitivity analysis was performed to determine the most impactful drug and formulation characteristics and therefore potential optimization parameters to achieve a high lung selectivity.

## Models

The mathematical model is introduced in a stepwise manner. First, the (sub)models describing the considered pulmonary PK processes are given. Next, the full PDE model it presented. The model parametrization is described in the Results section. Full details concerning derivations, numerical resolution, and additional model evaluations are given in S1 Appendix, as referenced below.

### Modeling of pharmacokinetic processes in the lung

#### Pulmonary particle deposition

Since orally inhaled drugs are deposited in the lungs within a single breath, pulmonary drug deposition was considered as an instantaneous rather than a time-dependent process. Pulmonary particle deposition was simulated with the MPPD software [21] according to the study design of each investigated study (i.e., for monodisperse particle size formulations as well as the specific particle size distributions of the Diskus and Turbohaler devices, respectively [22]). To simulate deposition patterns for asthmatic patients, who are characterized by a more central deposition compared to healthy volunteers [23, 24], we corrected the deposition patterns in healthy volunteers based on scintigraphy data reported in [25]. A full account of input parameters to predict the deposition patterns and the adaption for asthmatic patients is provided in S1 Appendix(Section 4). While the pulmonary deposition was considered different between healthy volunteers and asthmatic patients, other pulmonary PK processes and physiologic characteristics were assumed identical.

This procedure generated aerodynamic particle size- and lung generation-resolved deposition patterns. The aerodynamic particle size (the size of a water droplet experiencing the same aerodynamic forces as the considered particle) determines the deposition characteristics of the inhaled particles [26]. In contrast, the real (geometric) size of an inhaled particle is relevant for dissolution processes [27]. To convert aerodynamic to geometric particle sizes, which is more relevant for dissolution characteristics, we assumed a spherical shape of particles and considered the relationship

$$\begin{array}{c} d_{\text{geom}}=d_{\text{aero}}\sqrt{\frac{\rho_{\text{water}}}{\rho_{\text{substance}}}}, \end{array}$$

where *d*_aero_ and *d*_geom_ are aerodynamic and geometric particle diameters, respectively; *ρ*_water_ and *ρ*_substance_ denote density of water and the considered inhaled substance, respectively [28].

In a post-processing step, the (geometric) particle size- and lung generation-resolved deposition patterns were projected onto the computational grid, ensuring conservation of the number of molecules (full details are given in S1 Appendix, Section 2.5.1).

#### Mucociliary clearance

The mucociliary clearance process was parametrized based on a model for mucociliary clearance published by Hofmann and Sturm (see S1 Appendix, Section 1.2 for details) [10]. In agreement with clinical data, mucociliary clearance of undissolved particles only depends on the particle location, not on (geometric) particle size [26]:

$$\lambda_{\text{mc}}(x)=0.8791\frac{\text{cm}}{\text{min}}\cdot {\left(\frac{r^{\text{br}}(x)}{1\;\text{cm}}\right)}^{2.808},$$ (2)

where *r*^br^(*x*) represents the radius of the conducting airways at location *x*.

#### Pulmonary drug dissolution

The dissolution of particles in the pulmonary lining fluids was based on an adapted version of the Noyes-Whitney equation [27]:

$$\begin{array}{c} d\left(s,C_{\text{flu}}\right)=\frac{4\pi \;k_{\text{diss}}}{{\left(\frac{4}{3}\pi \right)}^{1/3}\;\rho }\cdot (1-\frac{C_{\text{flu}}}{C_{s}})\cdot s^{1/3}, \end{array}$$ (3)

where *s* denotes the particle volume, *ρ* the particle density, *C*_*s*_ the saturation solubility, *k*_diss_ = *D* ⋅ *C*_*s*_ the maximum dissolution rate (*D* = diffusivity), and *C*_flu_ the local concentration of dissolved drug in the lining fluid. A derivation of this equation from the Noyes-Whitney equation, assuming spherical particle geometry, is provided in S1 Appendix, Section 1.1. To represent the difference in fluid composition between conducting airways and the alveolar space, in particular in terms of fluid viscosity, different dissolution rate constants ($k_{\text{diss}}^{\text{br}}$/$k_{\text{diss}}^{\text{alv}}$) were assumed in these two regions, leading to dissolution models *d*^br^ and *d*^alv^, respectively.

#### Absorption into the lung tissues

After drug dissolution in the pulmonary lining fluids, the drug is absorbed through the airway epithelia into the lung tissue of the respective airway generation or the alveolar space. Based on reported negligible to tenfold lower albumin concentrations in epithelial ling fluids in the lung compared to plasma [29–31], the absorption rate is calculated assuming no drug binding in the lung lining fluids:

$$\begin{array}{c} k_{a}=P_{\text{app}}\cdot \text{SA}\cdot (C_{\text{flu}}-\frac{C_{\text{tis}}}{K_{\text{pu,tis}}}), \end{array}$$ (4)

where *k*_*a*_ denotes the absorption rate, *P*_app_ the effective permeability, SA the airway surface area, and *K*_pu,tis_ describes the lung-to-unbound plasma partition coefficient.

#### Systemic disposition

The systemic disposition models for both budesonide and fluticasone propionate were based on available literature information after intravenous administration and oral administration (to include the oral bioavailability of swallowed drug). In contrast to many less mechanistic PK models, the backflow of drug from the systemic circulation into the lung was mechanistically included in the PDE-based PK model.

### Partial differential equation model for orally inhaled drugs

#### Model equations

To mechanistically combine the considered pulmonary processes in the lung (lung deposition, mucociliary clearance, pulmonary dissolution, pulmonary absorption to the lung tissue and distribution between lung tissue and plasma), we adopted the framework of physiologically structured population models (PSPMs) [32]. In this class of PDE models, the time evolution of a density is described over a state space through a set of processes that modify the state.

To describe the fate of undissolved particles deposited in the lung, we considered (i) a PSPM with size and location structure in the conducting airways and (ii) a PSPM with size structure in the alveolar space. In these models, size *s* represents the geometric volume of particles, and location *x* (length unit) the position along all conducting airways, between trachea and terminal bronchioles. The state (*x*, *s*) of a particle is changed by mucociliary clearance (impacts on *x*) and pulmonary dissolution (impacts on *s*).

**Fig 2:**
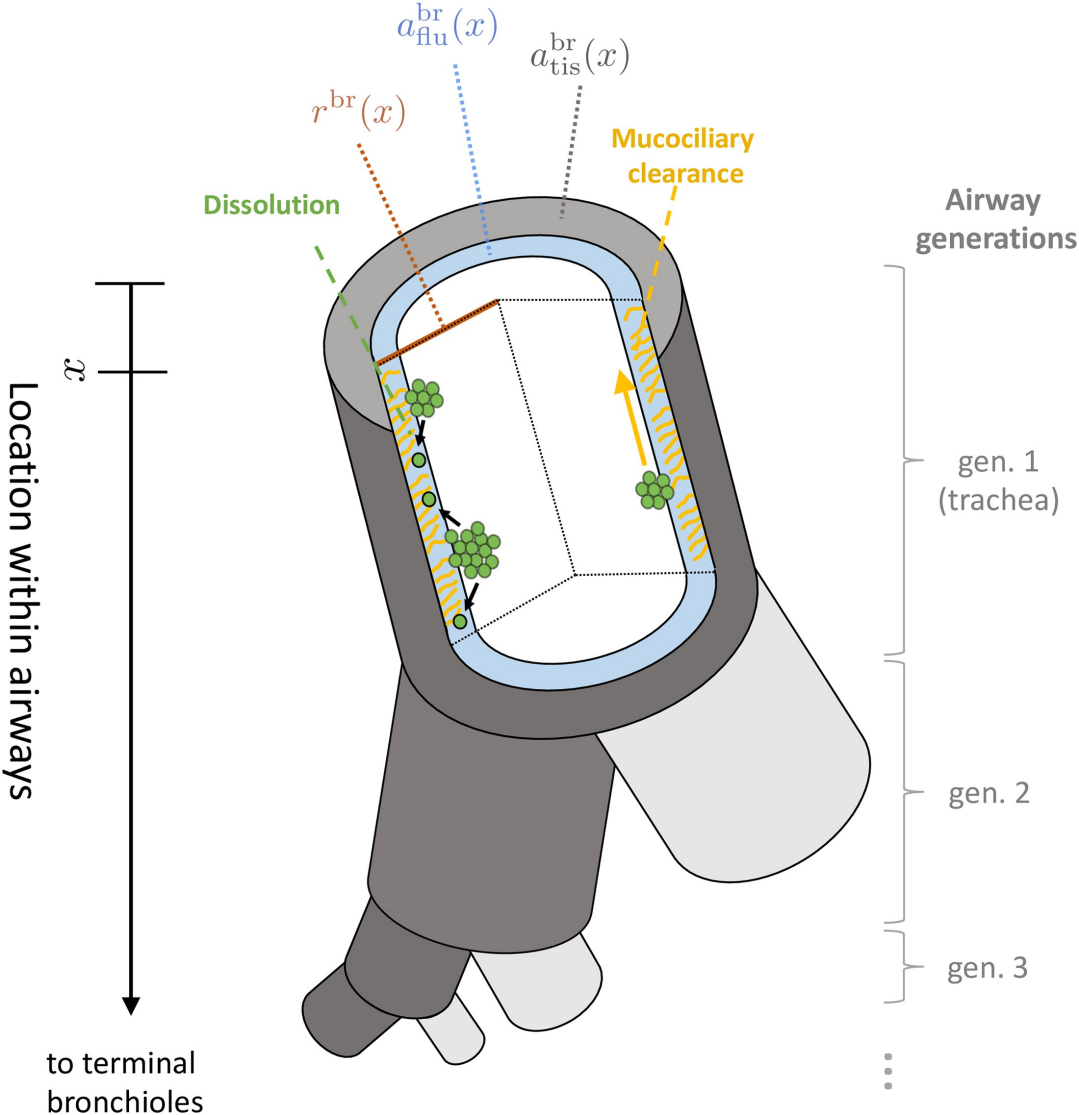
Geometry of and key processes in conducting airways represented in the mathematical model. A representative airway (dark grey) is considered in the mathematical model. At each location *x* (distance from throat) within this airway, we consider a cylindrical lung model, consisting of concentric layers of airway (with radius *r*^br^(*x*)), lung lining fluid (with cross-sectional area $a_{\text{flu}}^{\text{br}}\left(x\right)$), and lung tissue (with cross-sectional area $a_{\text{tis}}^{\text{br}}\left(x\right)$), respectively. Each drug particle (green) is characterized by its location and size. Over time, particles are moved upwards by mucociliary clearance (yellow) and dissolve into the airway lining fluid (black arrows).

The PSPMs were coupled to differential equations describing the PK of dissolved drug molecules in lung lining fluids and lung tissues (similar to [33]) and published systemic disposition kinetics [15]. The full set of equations is stated below, a simplified outline of the underlying geometry is provided in Fig 2 and a a detailed derivation of the full model from the separate PK processes is given in S1 Appendix (Sections 1.3-1.5).

For ease of legibility, the following abbreviations are used as sub-/superscripts in the equations: br (bronchial, i.e., conducting airways), alv (alveolar space), sys (systemic), sol (solid, i.e., undissolved), flu (fluid, i.e., dissolved), tis (lung tissue), ctr (central), per (peripheral), mc (mucociliary clearance).

The size- and location-structured PSPM for the density of inhaled particles in suspension in the conducting airways reads

$$\begin{array}{cc} & \partial_{t}\rho^{\text{br}}\left(t,x,s\right)+\partial_{x}[\lambda_{\text{mc}}\left(x\right)\rho^{\text{br}}\left(t,x,s\right)]-\partial_{s}[d^{\text{br}}\left(s,C_{\text{flu}}^{\text{br}}\left(t,x\right)\right)\rho^{\text{br}}\left(t,x,s\right)]=0\\ & \rho^{\text{br}}\left(0,x,s\right)=\rho_{0}^{\text{br}}\left(x,s\right)\;\;\text{(see}\;\text{S1}\;\text{Appendix,}\;\text{Sections}\;\text{2.5.1}\;\text{and}\;\text{4)} \end{array}$$

assuming zero inflow boundary conditions (i.e., no additional source of drug in the conducting airways after dosing). This PDE was complemented by differential equations for the concentration of dissolved drug in lining fluids and lung tissue at a particular airway location *x*:

$$\begin{array}{l} a_{\text{flu}}^{\text{br}}(x)\partial_{t}C_{\text{flu}}^{\text{br}}(t,x)=\underset{\text{dissolution}}{\underset{︸}{\int_{0}^{s_{\text{max}}}d^{\text{br}}(s,C_{\text{flu}}^{\text{br}}(t,x))\rho^{\text{br}}(t,x,s)\text{ds}}}-\underset{\text{tissue}\;\text{uptake}}{\underset{︸}{2\pi r^{\text{br}}(x)P_{\text{app}}(C_{\text{flu}}^{\text{br}}(t,x)-\frac{C_{\text{tis}}^{\text{br}}(t,x)}{K_{\text{pu,tis}}})}}\\ a_{\text{tis}}^{\text{br}}(x)\partial_{t}C_{\text{tis}}^{\text{br}}(t,x)=2\pi r^{\text{br}}(x)P_{\text{app}}(C_{\text{flu}}^{\text{br}}(t,x)-\frac{C_{\text{tis}}^{\text{br}}(t,x)}{K_{\text{pu,tis}}})-\underset{\text{systemic}\;\text{uptake}}{\underset{︸}{q^{\text{br}}(x)(\frac{\text{BP}C_{\text{tis}}^{\text{br}}(t,x)}{K_{\text{p,tis}}}-C_{\text{ctr}}^{\text{sys}}(t))}} \end{array}$$

both with zero initial conditions.

In the alveolar space, the size-structured PSPM for the density of inhaled particles in suspension reads

$$\begin{array}{cc} & \partial_{t}\rho^{\text{alv}}\left(t,s\right)-\partial_{s}[d^{\text{alv}}\left(s,C_{\text{flu}}^{\text{alv}}\left(t\right)\right)\rho^{\text{alv}}\left(t,s\right)]=0\\ & \rho^{\text{alv}}\left(0,s\right)=\rho_{0}^{\text{alv}}\left(s\right)\;\;\text{(see}\;\text{S1}\;\text{Appendix,}\;\text{Sections}\;\text{2.5.1}\;\text{and}\;\text{4)} \end{array}$$

Again, zero inflow boundary conditions were assumed (no additional source of drug in the alveolar space after dosing) and the PDE is complemented by differential equations for the concentration of dissolved drug in alveolar lining fluids and alveolar lung tissue:

$$\begin{array}{cc} V_{\text{flu}}^{\text{alv}}\frac{dC_{\text{flu}}^{\text{alv}}}{\text{dt}}\left(t\right) & =\int_{0}^{s_{\text{max}}}d^{\text{alv}}\left(s,C_{\text{flu}}^{\text{alv}}\left(t\right)\right)\rho^{\text{alv}}\left(t,s\right)\text{d}s-P_{\text{app}}\;{\text{SA}}^{\text{alv}}(C_{\text{flu}}^{\text{alv}}\left(t\right)-\frac{C_{\text{tis}}^{\text{alv}}\left(t\right)}{K_{\text{pu,tis}}})\\ V_{\text{tis}}^{\text{alv}}\frac{dC_{\text{tis}}^{\text{alv}}}{\text{dt}}\left(t\right) & =P_{\text{app}}\;{\text{SA}}^{\text{alv}}(C_{\text{flu}}^{\text{alv}}\left(t\right)-\frac{C_{\text{tis}}^{\text{alv}}\left(t\right)}{K_{\text{pu,tis}}})-Q^{\text{alv}}(\frac{\text{BP}\;C_{\text{tis}}^{\text{alv}}\left(t\right)}{K_{\text{p,tis}}}-C_{\text{ctr}}^{\text{sys}}\left(t\right)) \end{array}$$

with zero initial conditions.

The equations describing the PK in the conducting airways and the alveolar space are coupled through the systemic circulation:

$$\begin{array}{cc} V_{\text{ctr}}\frac{dC_{\text{ctr}}^{\text{sys}}}{\text{dt}}\left(t\right) & =\underset{\text{exchange}\;\text{with}\;\text{conducting}\;\text{airways}}{\underset{︸}{\int_{0}^{x_{\text{TB}}}q^{\text{br}}\left(x\right)(\frac{\text{BP}\;C_{\text{tis}}^{\text{br}}\left(t,x\right)}{K_{\text{p,tis}}}-C_{\text{ctr}}^{\text{sys}}\left(t\right))\text{d}x}}+\underset{\text{exchange}\;\text{with}\;\text{alveolar}\;\text{space}}{\underset{︸}{Q^{\text{alv}}(\frac{\text{BP}\;C_{\text{tis}}^{\text{alv}}\left(t\right)}{K_{\text{p,tis}}}-C_{\text{ctr}}^{\text{sys}}\left(t\right))}}\\ -Q^{\text{sys}}(C_{\text{ctr}}^{\text{sys}}\left(t\right)-C_{\text{per}}^{\text{sys}}\left(t\right))-\text{CL}\cdot C_{\text{ctr}}^{\text{sys}}\left(t\right)\\ V_{\text{per}}\frac{dC_{\text{per}}^{\text{sys}}}{\text{dt}}\left(t\right) & =Q^{\text{sys}}(C_{\text{ctr}}^{\text{sys}}\left(t\right)-C_{\text{per}}^{\text{sys}}\left(t\right)) \end{array}$$

The following expressions appear in these equations:

- *x* is the location within a prototypical airway, varying from 0 (trachea, corresponding to airway generation 1) to *x*_TB_ (terminal bronchioles, corresponding to airway generation 16).
- *s* is the geometric particle volume, varying between 0 and *s*_max_ (device- and formulation-specific maximum particle size deposited)
- *ρ*^br^/*ρ*^alv^ are the PSPM densities, with units $\frac{\text{number}\;\text{of}\;\text{particles}}{\text{mL}\;\cdot \;\text{cm}}$ and $\frac{\text{number}\;\text{of}\;\text{particles}}{\text{mL}}$, respectively
- $C_{\text{z}}^{\text{y}}$ is the concentrations of dissolved drug in lining fluid (z = flu) or lung tissue (z = tis) in a particular location of the conducting airways (y = br) or in the alveolar space (y = alv)
- *d*^br^ / *d*^alv^ are the dissolution rates in conducting airways / alveolar space, depending on particle size *s* and concentration of already dissolved drug
- λ_mc_ is the mucociliary clearance in the conducting airways, assumed to depend only on location *x*, not on (geometric) particle size *s*.
- *P*_app_ is the apparent permeability of the drug
- SA^alv^ is the surface area of the alveolar space
- *r*^br^(*x*) is the airway radius (including lining fluid) at location *x* (see Fig 2)
- *K*_p,tis_ / *K*_pu,tis_ are the lung-to-plasma and lung-to-unbound plasma partition coefficients, respectively
- BP is the blood-to-plasma ratio of the drug
- $a_{\text{flu}}^{\text{br}}\left(x\right)$ / $a_{\text{tis}}^{\text{br}}\left(x\right)$ is the cross-sectional area of lung lining fluid / lung tissue at location *x* within the conducting airways
- *q*^br^(*x*) is the location-resolved blood flow (see section Model parametrization below)

#### Numerical resolution

To solve the mathematical model numerically, we employed an upwind discretization of the PSPMs [34] together with an implicit discretization of all linear processes (MCC, absorption, systemic processes) [35, 36]. The fluxes across PDEs (mucociliary elevator and dissolved / absorbed drug) were discretized ensuring that all conservation laws were fulfilled at the discrete level. The discretized model and all analyses were implemented in MATLAB R2018b [37]. A full description of the discretization scheme is given in S1 Appendix (Section 2) and the MATLAB implementation is provided as S1 File.

## Results

### Key findings from literature review

As a first step, PK studies for both budesonide and fluticasone propionate were identified. These drugs were selected as they represent the most studied inhaled drugs for which the interplay between pulmonary deposition, pulmonary dissolution, mucociliary clearance, as well as pulmonary absorption has been systematically discussed [2, 24]. In total, ten different clinical PK studies on these drugs were identified (see S1 Table).

After reviewing all PK studies, we identified two important aspects. First, the area under the curve reported by Usmani et al. [38] could not be reproduced considering the systemic clearance for fluticasone propionate reported by Mackie et al. [39]. Even in the most extreme and certainly unrealistic assumptions, namely with 100% of inhaled drug particles deposited in the lungs and no mucociliary clearance, the systemic AUC would still be at least 25% lower than reported (see calculation in S1 Appendix, Section 3.3).

Second, there is a considerable between-study variability in reported (dose-normalized) systemic drug exposure (same drug, comparable dose, comparable patient population, same inhalation device). For example, both Möllmann et al. [40] and Harrison and Tattersfield [41] investigated the systemic PK after budesonide inhalation with the Turbohaler for slightly different doses of 1000 *μ*g and 1200 *μ*g. The reported dose-normalized C_max_ and AUC_0-Inf_ values varied by more than twofold. In contrast, the relative shape of the PK profiles, which are not dependent on the absolute plasma concentrations, were in good agreement between both studies. A full summary of exposure metrics is given in S1 Table.

Based on these findings, we decided that predicting the absolute plasma concentrations of one single selected PK study is not meaningful or could even result in a selection bias. Instead, PK studies with multiple study arms, which allow for a direct within-study comparison of different PK profiles, were considered (i.e., studies with only a single investigated drug, a single inhaled particle size and a single investigated population were not included). A short overview of the reviewed PK studies, including a comment on why specific studies were considered, can be found in S1 Table. In summary, the selected PK studies comprised the following aspects relevant for model building and model evaluation: (i) the lung retention profiles for insoluble particles [26], (ii) the impact of different particle sizes on the systemic PK [38], (iii) different systemic PK profiles after inhalation of either budesonide or fluticasone propionate [40, 41], and (iv) different systemic PK profiles between healthy volunteers and asthmatic patients [41].

### Model parametrization

The PDE-based model was not adapted to individual studies, i.e., no (pharmacokinetic) parameters were estimated based on the studies which were used for model evaluation. Instead, the pulmonary part of the PDE model was fully parametrized based on physiological and drug-specific *in vitro* data (*a priori* predictions).

Both pulmonary drug deposition and mucociliary clearance were considered as drug-independent generic processes based on particle size and airway characteristics alone, not requiring any drug-specific parameters. Drug-specific parameters, such as the maximum dissolution rate (*k*_diss_), as well as drug solubility in pulmonary lining fluids were either based on literature information or in-house data on *in vitro* dissolution and solubility. No direct comparison between alveolar and mucus dissolution kinetics could be retrieved from literature or in-house data. Therefore, a 5-fold decrease of *k*_diss_ in the conducting airways compared to the alveolar space was assumed for all model-based simulations. A comprehensive list of parameter values is given in Table 1 (physiological parameters) and Table 2 (drug-specific parameters).

**Table 1:** Physiological parameters^#1^.

Parameter	Symbol(s)	Value
Perfusion of conducting airways	*Q*^br^	7.8 L/h ^#2^
Perfusion of alveolar space	*Q*^alv^	312 L/h ^#3^
Bronchial tissue volume	$V_{\text{tis}}^{\text{br}}$	144 mL ^#4^
Alveolar tissue volume	$V_{\text{tis}}^{\text{alv}}$	388 mL ^#4^
Alveolar fluid volume	$V_{\text{flu}}^{\text{alv}}$	36 mL [42]
Alveolar surface area	${\text{SA}}_{\text{flu}}^{\text{alv}}$	130 m^2^ [43]
Location-resolved parameters	*r*^br^, $a_{\text{flu}}^{\text{br}}$, $a_{\text{tis}}^{\text{br}}$, *q*^br^	see main text

^#1^ Physiological parameters were assumed identical for healthy volunteers and asthmatic patients. Only deposition patterns were corrected for asthmatic patients.

^#2^ calculated based on 2.5% of cardiac output [44]

^#3^ equal to cardiac output, taken from [45, Table 22]

^#4^ computed from lung tissue weight of 532 g [45], assuming a tissue density of 1 g/mL and 27% central/73% peripheral lung tissue weight fraction as in [33, Supplement].

Summary of physiological parameters obtained from literature.

**Table 2:** Drug-specific parameters.

Parameter	Symbol	Fluticasone propionate	Budesonide
Central volume of distribution	*V*_ctr_	31 L [15]	100 L [15]
Peripheral volume of distribution	*V*_per_	613 L [15]	153 L [15]
Clearance	CL	73 L/h [15]	85 L/h [15]
Intercompartmental clearance	*Q*^sys^	55.2 L/h [15]	1701 L/h [15]
Oral bioavailability (of swallowed drug)	*F*_oral_	0% [15]	11% [15]
Absorption rate constant from GI tract	*k*_*a*_	–	0.45 1/h [15]
Fraction unbound in plasma	*f*_u,plasma_	1.16%^#1^	16.1%^#1^
Lung:plasma partition coefficient	*K*_p,tis_	2.47^#2^	8 [46]
Permeability	*P*_app_	92.6 ⋅ 10^−6^ cm/s^#1^	5.33 ⋅ 10^−6^ cm/s [47]
Blood:plasma ratio	BP	1.83^#1^	0.8 [48, 49]
Molecular weight	MW	500.57 g/mol	430.53 g/mol
Density	*ρ*	1.43 mol/L	3.02 mol/L
Solubility	*C*_*s*_	12.0 *μ*M^#1^	69.8 *μ*M^#1^
Maximum dissolution rate	$k_{\text{diss}}^{\text{alv}}$	$6.17\cdot 10^{-5}\;\frac{\text{nmol}}{\text{cm}\cdot \text{min}}$^#3^	$3.3\cdot 10^{-4}\;\frac{\text{nmol}}{\text{cm}\cdot \text{min}}$^#3^
Inhalation device-specific parameters	see S1 Appendix, Section 4		

^#1^ in-house data: fraction unbound was determined with an *in vitro* binding assay as described in [50], permeability was determined based on an *in vitro* permeability assay with Calu cells, with assay conditions as described for MDCK II cells in [50]. The *in vitro* assay setup for determining Blood:Plasma ratio and drug solubility in surfactant-containing media is described in S1 Appendix, Section 5.

^#2^ calculated based on *f*_u,plasma_ (in-house data) and rat lung slice binding [51]

^#3^ determined from *in vitro* dissolution data from [52] (see S1 Appendix, Section 3.1 for full details).

For both drugs, two-compartment systemic PK models proposed in the literature were used.

To achieve a location-resolved parametrization of the conducting airways, we used generation-specific anatomical data of the conducting airways from [53], namely the length *l*(*g*) and radius *r*(*g*) of each airway generation *g*. From these values, location-resolved blood flows and cross-sectional lining fluid and lung tissue areas were calculated by assuming the following:

- Using length of airway generations, we determined a continuous representation *r*^br^(*x*) of the airway radius by linear interpolation between airway centerpoints.
- We assessed literature data on lining fluid height $h_{\text{flu}}^{\text{br}}\left(x\right)$ for different airway generations and found an appropriate linear location-to-height of lining fluid-relationship (see S1 Appendix, Section 3.2, for details). Using the cylindrical geometry assumption depicted in Fig 2, $a_{\text{flu}}^{\text{br}}\left(x\right)$ could be determined from $h_{\text{flu}}^{\text{br}}\left(x\right)$ and the airway radius *r*^br^(*x*) via
  $$a_{\text{flu}}^{\text{br}}(x)=\pi (r^{\text{br}}{\left(x\right)}^{2}-{\left(r^{\text{br}}(x)-h_{\text{flu}}^{\text{br}}(x)\right)}^{2}).$$
- We assumed the cross-sectional area of conducting airway tissue $a_{\text{tis}}^{\text{br}}\left(x\right)$ to be proportional to cross-sectional lining fluid area $a_{\text{flu}}^{\text{br}}\left(x\right)$, with proportionality constant determined by the known total tissue volume of the central lung $V_{\text{tis}}^{\text{br}}$, i.e., via the relation $\int_{0}^{x_{\text{TB}}}a_{\text{tis}}^{\text{br}}\left(x\right)dx=V_{\text{tis}}^{\text{br}}$.
- We assumed a homogeneous perfusion of drug tissue within the conducting airway tissue, i.e., a location-resolved blood flow *q*^br^(*x*) proportional to $a_{\text{tis}}^{\text{br}}\left(x\right)$ and matching the total blood flow in the central lung *Q*^br^, i.e. such that $\int_{0}^{x_{\text{TB}}}q^{\text{br}}\left(x\right)dx=Q^{\text{br}}$.

We emphasize that the pulmonary PDE model was fully parametrized based on *in vitro* and physiological data, not fitted to the clinical data described in section Model evaluation below. A single adaptation was done based on physiological reasoning since no quantitative literature information could be retrieved, namely a 5-fold decrease of dissolution rate in the conducting airways compared to the alveolar space. The reason for this adapted dissolution rate constant is that the epithelial lining fluid in the conducting airways –the mucus– contains a lower concentration of surfactants (which facilitate dissolution), compared to the alveolar lining fluid. In addition, the upper layer of the mucus is characterized by a high viscosity, which can also lead to a slower dissolution in comparison to the alveolar space.

### Model evaluation

**Fig 3:**
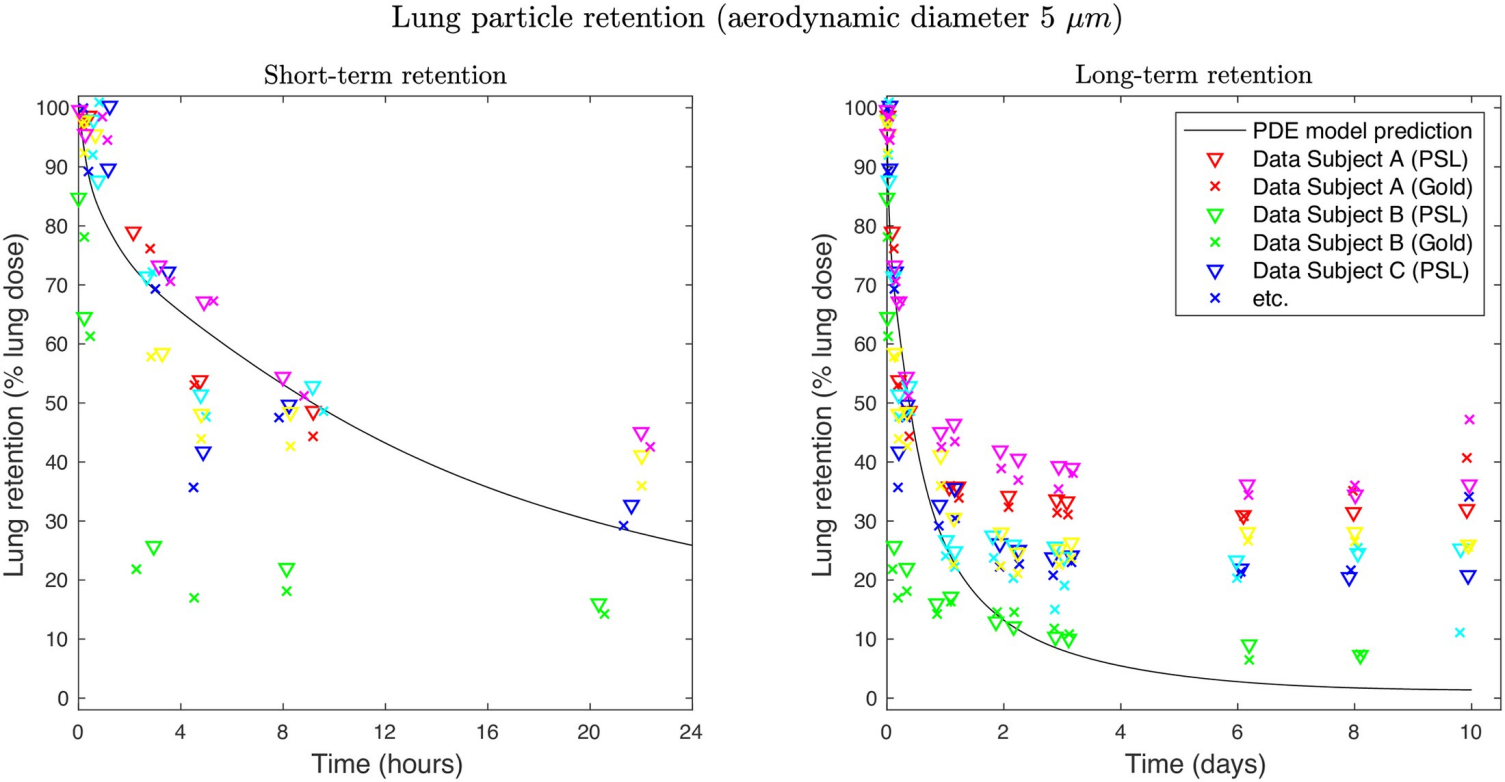
Pulmonary retention profiles of inhaled insoluble particles. Pulmonary retention of inhaled monodisperse 5 *μ*m-sized (aerodynamic diameter) gold and polystyrene (PSL) particles. The amount retained is described as a fraction of the initially deposited lung dose. Left: retention-time profile over 24 h, right: retention-time profile over 10 days. Data points: digitized data from six subjects from [26]; solid line: model-based predictions of lung retention.

The mathematical model was evaluated in a stepwise approach. The first evaluation of the PDE-based inhalation PK model was based on a simulation of inhaled gold / polystyrene particles. As these particles do not dissolve in the pulmonary lining fluids, the interplay of deposition and mucociliary clearance can be evaluated independent of other pulmonary PK processes such as pulmonary dissolution or drug absorption. The initial particle retention was well described with 53% of the deposited particles retained over 8 h (observed median at 8–10 h: 48.5%) and 26% retained over 24 h (observed median at 20–26 h: 34%), see Fig 3 (left). However, the retention after 48 h was underpredicted, i.e. the data indicated a fraction of 7–36% not being cleared from the lung, whereas the simulation indicated less than 5% retention (Fig 3, right). The squared correlation coefficient between observed and model-predicted retention (all time points pooled) was *r*^2^ = 0.86 (see S4 Fig).

After evaluating the interplay of pulmonary deposition and mucociliary clearance, the pulmonary dissolution process was evaluated based on data from inhaled monodisperse drug formulations. In this evaluation step, the systemic PK of 1.5, 3, and 6 *μ*m-sized particles (aerodynamic diameter) were simulated for the slowly dissolving inhaled drug fluticasone propionate. Simulation results were compared to the determined AUC_0-12h_, C_max_, and T_max_ published by Usmani et al. [38]. As explained above, the absolute exposure metrics stated in the publication could not be reproduced. Rather than through goodness of prediction of *absolute* exposure measures, we therefore evaluated the model by comparing the *relative* change of exposure metrics across the three considered particle sizes. Of the model-predicted exposure metrics AUC_0-12h_ and C_max_, 67% were within 2-fold and 83% within 3-fold of the reported ratios (compare Table 3). The predicted 1.5 *μ*m: 3 *μ*m T_max_ ratio matched the experimental data well, however the other predicted T_max_ ratios showed larger discrepancies due to a predicted very flat concentration-time profile for 6 *μ*m particles.

**Table 3:** Evaluation of model predictions for different particle sizes.

Exposure metric ratio	AUC_0-12h_		C_max_		T_max_	
	Data	Model	Data	Model	Data	Model
1.5 *μ*m: 3 *μ*m	1.04	1.65	1.52	4.23	0.40	0.52
1.5 *μ*m: 6 *μ*m	4.16	4.92	5.00	20.7	0.26	0.09^#1^
3 *μ*m: 6 *μ*m	4.01	2.98	3.27	4.90	0.63	0.17^#1^

^#1^ For 6 *μ*m particles, the predicted concentration-time profile was very flat, resulting in a late T_max_ and therefore low 1.5 *μ*m: 6 *μ*m and 3 *μ*m: 6 *μ*m T_max_ ratios.

Comparison of model-predicted and reported PK between three different inhaled monodisperse particle formulations of fluticasone propionate, with aerodynamic diameters of 1.5, 3, and 6 *μ*m, respectively [25]. Due to uncertainty in reported absolute PK parameter readouts, the ratios between both listed particle sizes are reported instead. For example, the 1.5 *μ*m-sized particles yielded a 4.16 fold higher measured AUC_0-12h_ in comparison to the 6 *μ*m-sized particles, whereas the model-based prediction resulted in 4.92 fold higher AUC_0-12h_.

**Fig 4:**
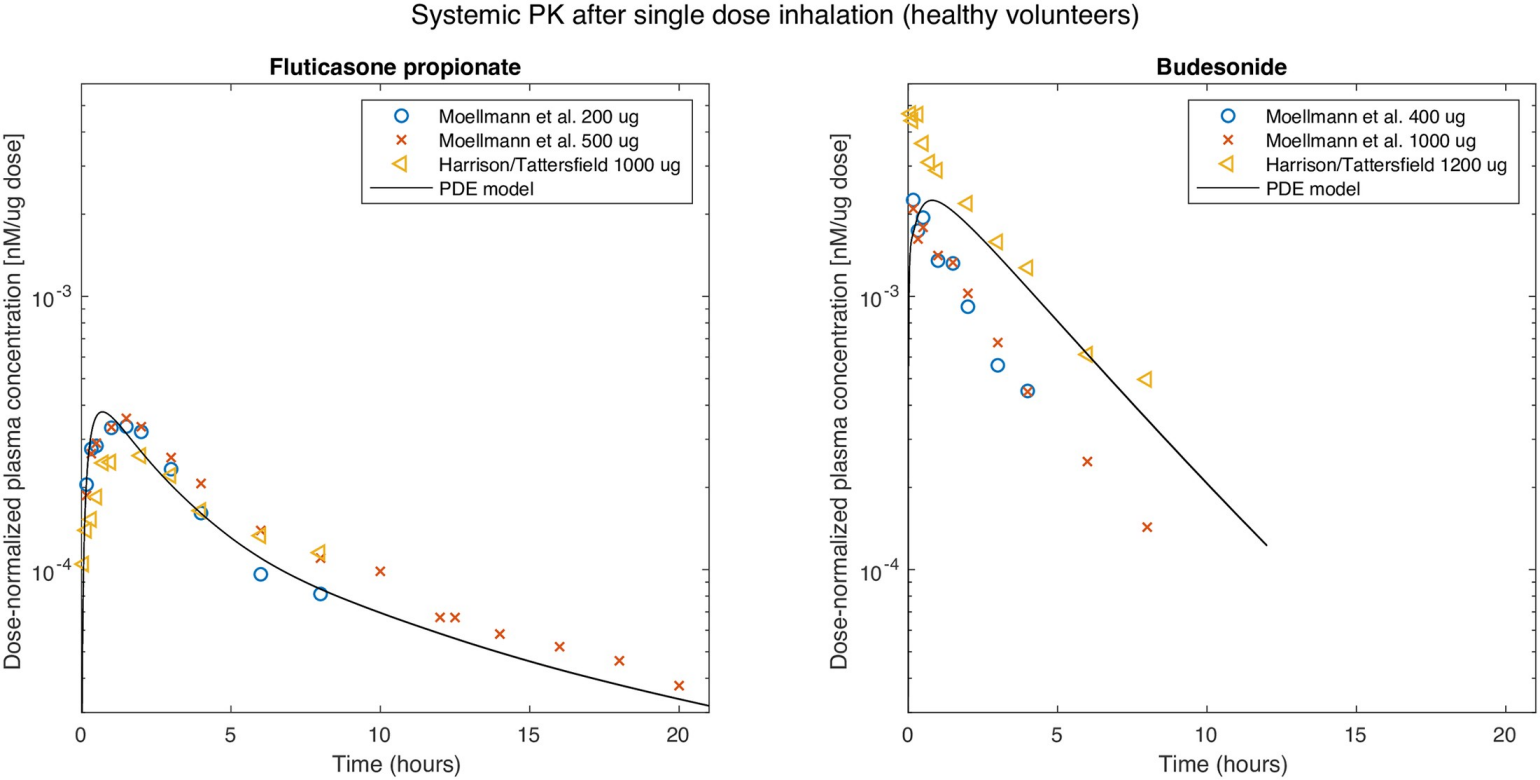
Pharmacokinetics after drug inhalation of clinical formulations. Plasma concentration-time profiles after drug inhalation of fluticasone propionate inhaled with the Diskus inhalation device (left panel) and budesonide inhaled with the Turbohaler inhalation device (right panel). Data points: digitalized raw data from [40, 41], solid lines: PDE-model based predictions for 200, 500, and 1000 *μ*g doses of fluticasone propionate and 400, 1000, and 1200 *μ*g doses for budesonide (due to an almost dose-linear PK, model predictions overlap).

As a last step of the PDE model evaluation, systemic PK profiles of fluticasone propionate and budesonide were simulated for both healthy volunteers and asthmatic patients, the only assumed difference between both populations being a more central particle deposition in asthma patients (see deposition profiles in S2 Fig). For fluticasone propionate inhaled by healthy volunteers with the Diskus device, a C_max_ of 0.38 nM per mg dose, an AUC_0-12h_ of 1.8 nM ⋅ h per mg dose, and a T_max_ after 41 min were predicted. For budesonide (Turbohaler), dissolution as well as absorption to the systemic circulation were predicted to be faster compared to fluticasone propionate, with a C_max_ of 2.2 nM per mg dose; T_max_ was similar and AUC_0-12h_ larger (10 nM ⋅ h per mg dose). The model-predicted PK profiles for fluticasone propionate and budesonide in comparison to observed clinical data from healthy volunteers [40, 41] are displayed in Fig 4 and S5 Fig. For fluticasone propionate, the dose-normalized data from literature were in agreement, and the simulation results closely matched these data (*r*^2^ = 0.94). For budesonide, there was a between-study, but not within-study discrepancy between reported dose-normalized concentration-time profiles; model predictions were well within the reported range (*r*^2^ = 0.76). The discrepancy in the data was not explainable by dose-nonlinear PK, since a 2.5-fold dose change in [40] did not impact on the normalized profiles. Of note, the model-predicted dose-normalized concentration-time profiles based on these scenarios all overlapped, which agrees with the clinically observed absence of dose-dependent pharmacokinetics in plasma for both fluticasone propionate and budesonide.

The same simulations for asthmatic patients resulted in lower systemic exposure. For fluticasone propionate, 28% of the initially deposited lung dose was predicted to be eliminated via mucociliary clearance in healthy volunteers, compared to 53% in asthmatic patients due to the more central particle deposition. For budesonide, 6% and 29% of the initially deposited lung dose were predicted to be eliminated via mucociliary clearance in healthy volunteers and asthmatic patients, respectively. A comparison of model-predicted and clinically observed differences between healthy volunteers and asthmatic patients is given in Table 4. For fluticasone propionate, simulations were in good agreement with clinical data, whereas for budesonide, the model overpredicted the impact of asthma on AUC_0-12h_ and C_max_, which was reported as non-significant in [41] (i.e., ratios of exposure metrics close to 1). However, the model-predicted stronger disease effect for fluticasone propionate compared to budesonide –in terms of a larger AUC_0-12h_ ratio– was in agreement with the clinical data.

**Table 4:** Evaluation of model-predicted PK differences between healthy volunteers and asthmatic patients.

Healthy:asthmatic ratio for substance	AUC_0-12h_		C_max_		T_max_	
	Data	Model	Data	Model	Data	Model
Fluticasone propionate	1.76	1.64	1.67	1.48	1	1.05
Budesonide	0.88	1.43	1.07	1.51	NA^#1^	1.05

^#1^ since the reported T_max_ values both corresponded to the first observed time point, no meaningful statement about T_max_ ratios can be made.

Comparison of model-based and literature-reported PK difference between healthy volunteers and asthmatic patients. Data are taken from [41] (1000 *μ*g fluticasone propionate with Diskus / 1200 *μ*g budesonide with Turbohaler). Ratios larger than 1 indicate higher values in healthy volunteers, whereas ratios smaller than 1 indicate higher values in asthmatic patients. NA, not available.

### Sensitivity analysis

As a last step of the analysis, a sensitivity analysis was applied to the evaluated PDE model to determine the most impactful parameters (among formulation-dependent, physiological, and drug-specific parameters) on the following PK readouts:

(i) AUC_0-24h_ in conducting airway tissue,
(ii) the average concentration in the conducting airway tissues after 24 h (which is supposed to correlate with long-lasting efficacy of an inhaled drug), and
(iii) lung selectivity, which is expressed as a ratio between the pulmonary AUC (in conducting airways) and the systemic AUC.

**Fig 5:**
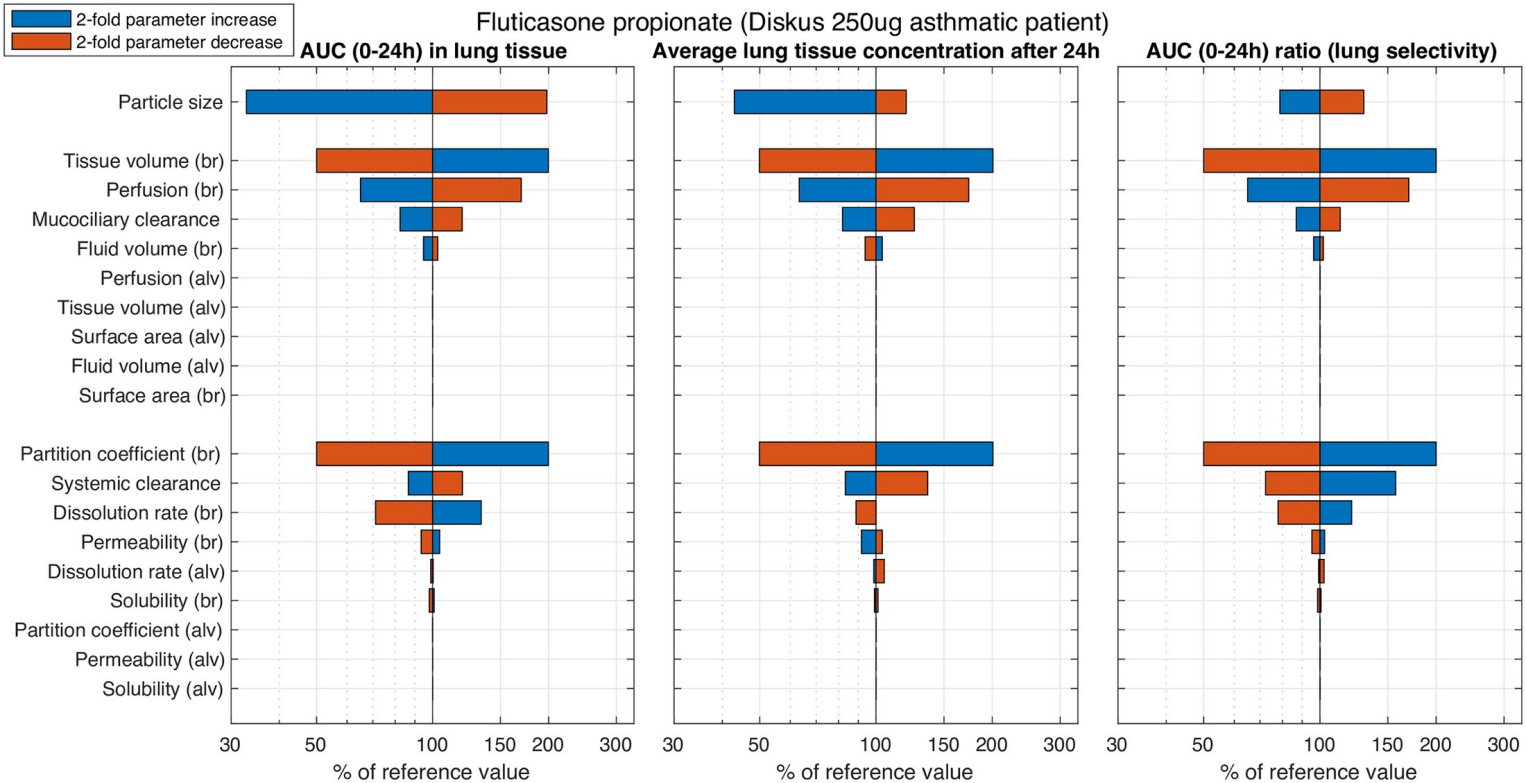
Results of the performed sensitivity analysis for fluticasone propionate. For each of three different exposure measures readouts (AUC, *C*_24_, and lung selectivity), the impact of a 2-fold increase (blue) and decrease (red) are depicted for the formulation parameter particle size (top bar) and a set of physiological (middle bars) and drug-dependent parameters (bottom bars). The larger a bar, the stronger the impact of the varied parameter on the respective PK readout.

This last quantity is supposed to provide a metric of local efficacy weighed against systemic safety, which is an important optimization criterion for inhaled drugs. As the relevance of an input parameter can depend on the complete set of the initial input parameters, the sensitivity analysis was performed starting with the parameters for (i) a 250 *μ*g fluticasone propionate dose (see Fig 5) and (ii) a 800 *μ*g budesonide dose (see S1 Fig), both representing approved doses [54, 55].

Overall, the order of impactful parameters only differed marginally for the different exposure metrics and different drugs. A more than 50% change was observed for tissue volume, tissue partition coefficient, perfusion, dissolution rate and systemic clearance. Particle size had a considerable impact for fluticasone propionate, and less for budesonide. For fluticasone propionate, the impact of drug solubility in the airway lining fluids was negligible, whereas for budesonide, although being the more soluble drug, a relevant impact was predicted since lining fluid concentrations approached the solubility (see S3 Fig). Other parameters, such as lining fluid volume and all physiological parameters related to the alveolar space were characterized by negligible impact on the exposure metrics. The impact of deviating the model parameters by a 2-fold increase and 2-fold decrease was typically antithetical. As a notable exception, the dissolution rate in the conducting airways resulted in lower lung tissue concentration after 24 h for fluticasone propionate, regardless of whether the dissolution rate was increased or decreased 2-fold.

## Discussion

The pulmonary pharmacokinetics of orally inhaled drugs are highly complex as pulmonary deposition, pulmonary dissolution, mucociliary clearance, and pulmonary absorption create a complex interplay. Consequently, defining adequate optimization parameters for orally inhaled drugs remains challenging. To adequately capture and mechanistically predict the complex interplay of all pulmonary PK processes and to identify optimization parameters, a PDE-based mechanistic PK framework was developed.

To build sufficient trust into a pulmonary PK model to use it for identification of optimal drug characteristics, an adequate and systematic model evaluation is a prerequisite. However, previous mechanistic modeling attempts, most noticeably the ones by Caniga et al. [56] and Boger et al. [20], lack such a thorough evaluation. Indeed, the approach by Caniga et al., differentiating between airways and alveolar space albeit less mechanistically than in the here-presented model, was evaluated for inhaled mometasone [56] and more recently for additional fast dissolving drugs (formoterol, salbutamol, and budesonide) [57]. However, these drugs would not provide the same insights into the pulmonary interplay of deposition, mucociliary clearance, and dissolution as the slowly dissolving drug fluticasone propionate. An adequate prediction quality for healthy and diseased populations, different particle sizes, slowly dissolving drugs or even insoluble particles remains to be demonstrated.

A PDE model published by Boger et al. mechanistically included all pulmonary PK processes [20]. However, this model was based on a hypothetical drug, and while most of the characteristics of this hypothetical drug can be considered reasonable, such as a *K*_p,lung_ of 4.9 or the oral bioavailability of 20%, no *in vitro* assays can be used to characterize drug characteristics such as permeability, dissolution kinetics, and solubility. Therefore, and since a model evaluation against clinical data is not feasible for a hypothetical drug, no assessment of the model’s predictive capacities was made.

Therefore, the here-presented model represents –to the best of our knowledge– the first systematically evaluated and publicly available mechanistic pulmonary PK model. First, to evaluate the mechanistic implementation of the mucociliary clearance, model-based lung retention profiles were compared to the pulmonary retention of insoluble gold and polystyrene particles. Short-term particle retention was adequately predicted, whereas long-term retention was underpredicted. One potential explanation could be that even with small inhaled volumes, a fraction of the inhaled particles can deposit in alveoli e.g., due to asymmetry in airway branching. Since drug deposited in the alveolar space is not cleared by the mucociliary clearance, this would result in higher long-term lung retention than predicted. Other explanations might be that the mucus flow is not uniform in the conducting airways with more “static” mucus regions, in which particles are slowly cleared [58]. Or alternatively that the gel phase of the mucus is not continuous and particles, which are sinking deeper into the mucus (due to the “missing” gel phase) are subsequently cleared slower [59]. A more detailed discussion of this phenomenon is provided by Smith et al. [26]. Even though there are still discussions ongoing on the details of the mucociliary clearance, there is a high evidence for a slower mucociliary clearance in peripheral airways and absence of mucociliary clearance in the alveolar space, which is adequately represented in our mechanistic pulmonary PK model. Furthermore, as long-term pulmonary drug retention should not be relevant for orally inhaled drugs, the identified discrepancy was considered acceptable.

Second, to evaluate the mechanistic implementation of the interplay of particle deposition, mucociliary clearance, and pulmonary drug dissolution, the PK of fluticasone propionate for different monodisperse particles (1.5, 3, and 6 *μ*m aerodynamic diameter) were predicted and compared to published data [38]. It has to be stated that the reported absolute exposure metrics could not be reproduced. However, they appear extraordinarily high and could not be reached even if the provided dose had been administered intravenously. Nevertheless, the publication by Usmani et al. contains a unique data set, and therefore we still considered the dataset, but by comparing the relative, not absolute, differences between the predictions for different particle sizes. The trends in this dataset, namely a decrease in exposure and a delayed uptake with increasing particle size, were well predicted by the model. Quantitative mismatches of the predicted particle size effect might have been caused partially also by high inter-occasional variability (which can be relevant for group sizes of *n* = 15). Furthermore, the sampling scheme underlying the calculation of exposure metrics was not reported in [38] and might have an impact in particular on C_max_ and T_max_ values. Based on this evaluation, we considered the model-based predictions for the varying particle size effect as good.

Third, the modeling framework was used (without estimating additional input parameters) to simultaneously predict the PK of both fluticasone propionate and budesonide. For both drugs, plasma concentration-time profiles in healthy volunteers were very well predicted. In addition, the difference in pharmacokinetics between healthy volunteers and asthmatic patients was well predicted for fluticasone propionate. In contrast, the impact of disease on the PK of budesonide was overpredicted, i.e. in asthmatic patients more drug was predicted to be cleared by mucociliary clearance before it could be absorbed. One potential reason for this discrepancy is the strongly increased deposition of drug particles in the first airway generations assumed for asthmatic patients (see S2 Fig), which resulted from the assumption that the deposition probability across all airway generations is increased to a similar extent by local airway obstructions. This assumption contrasts with literature discussing qualitatively that airway obstructions in asthma are located more peripherally in the conducting airways (in higher airway generations) [60] and therefore the deposition would increase in more peripheral conducting airways rather than in the trachea and first airway generations (as can be seen in the imaging data in [25]). Unfortunately, we are not aware of quantitative data or deposition models based on such data, which would allow to better account for differences between healthy volunteers and asthmatic patients. Therefore, we were unable to integrate a more adequate representation into our mechanistic model. In comparison to pulmonary deposition, even less quantitative information is available on altered physiology in asthmatic patients. Some studies indicated e.g., thicker airway walls or peripheral airway closing, as well as airway remodeling in asthmatic patients [61, 62]. As airway remodeling in asthma was discussed to increase over time as a result of the inflammation, it was discussed more relevant for severe asthma [63]. Therefore, and based on missing quantitative data, we assumed that the physiological parameters (e.g., pulmonary perfusion and pulmonary surface areas) are identical between healthy volunteers and (mild to moderate) asthmatic patients. For severe asthma as well as other (restrictive) pulmonary diseases, adapting the lung physiology might however be necessary.

Based on the overall good agreement between the predictions and observed clinical data, we consider the here-published PDE-based PK model as the currently best-evaluated mechanistic model for orally inhaled drugs. However, even this mechanistic PK model still represents a simplification of reality and only includes the above-mentioned pulmonary PK processes; macrophage clearance as well as pulmonary metabolism were assumed not relevant. For some specific inhaled drugs, this assumption might not hold true. For example, pulmonary metabolism was discussed to be of importance for inhaled macromolecules (e.g., insulin [64, 65]). Macrophage clearance from the alveolar space to the conducting airways was characterized by a very long half-life of 35–115 days [66, 67]. Consequently, compared to pulmonary absorption and dissolution kinetics of most inhaled drugs, macrophage clearance is expected to be negligible. Furthermore, the considerable between-study variability in reported data has to be kept in mind when judging the model evaluation accuracy. To recognize all of these assumptions, to understand their potential impact on the pulmonary PK, and finally to adequately apply the here presented model framework, a sound understanding of respiratory drug delivery remains essential.

As a last step of the presented analysis, we investigated the most relevant optimization parameters for orally inhaled drugs. To this end, we performed a model-based sensitivity analysis to identify the most impactful model parameters on pulmonary exposure metrics. The pulmonary AUC was considered as a surrogate for pulmonary efficacy and the average concentration in the conducting airways after 24 h was considered a surrogate for the effect duration of an inhaled drug. Finally yet importantly, the ratio between pulmonary and systemic exposure was considered as a surrogate for lung selectivity of an inhaled drug (i.e. the larger the ratio, the better the lung selectivity).

An impactful formulation-dependent model parameter was the particle size distribution of the inhaled fluticasone propionate formulation. This might not be surprising as the particle size simultaneously affects various pulmonary PK processes, i.e., larger particles deposit more centrally, dissolve slower and therefore a higher fraction of drug would be cleared by the mucociliary clearance. As a result, model-based predictions for larger particles indicated less lung exposure, shorter drug residence times in the lung, as well as a lower lung selectivity. In contrast, smaller fluticasone propionate particles would improve all exposure metrics. In conclusion, the model-based prediction framework indicates that reducing the particle size for inhaled fluticasone propionate would be a reasonable optimization parameter. However, this optimization parameter was predicted relevant only for fluticasone propionate. In contrast, the sensitivity analysis predicted no relevant impact of the particle size to be expected for a drug like budesonide.

Impactful drug-specific optimization parameters for both drugs were (i) the lung partition coefficient, (ii) the systemic clearance, and (iii) the dissolution rate. An increase in the pulmonary partition coefficient, which indicates an increase in the pulmonary tissue affinity, was already previously suggested as an optimization parameter for lung selectivity [17, 68, 69]. This parameter however has to be considered carefully as a high tissue affinity / binding also would decrease the free pulmonary concentration. The systemic clearance had low impact on the pulmonary drug concentrations, but a higher systemic clearance provided a better lung selectivity. Therefore, especially for drugs with a critical systemic safety profile increasing the systemic clearance can be considered meaningful. In agreement, the relevance of a high systemic clearance to reduce systemic adverse effects for orally inhaled drugs was previously discussed [17, 70]. The pulmonary dissolution rate for fluticasone propionate already seems to be nearly optimal to achieve a long-lasting efficacy, which would be a good property for a once-daily administered drug. An additional decrease in the dissolution kinetics was predicted to rather decrease the long-lasting pulmonary exposure. This finding is in agreement with recent observations that increasing the tissue affinity might be a better strategy to prolong the efficacy compared to slow dissolution [71]. Interestingly, while the dissolution rate constant can still be considered an optimization criterion, the solubility in the airway lining fluid was not impactful for fluticasone propionate. This underlines that actually the dissolution rate and not the solubility might be important for pulmonary drug administration. For budesonide, which is characterized by faster dissolution kinetics compared to fluticasone propionate, the solubility was as important as the dissolution rate constant. The reason is that for budesonide, four parameters simultaneously increased local drug concentrations in the epithelial lining fluids: (i) a higher inhaled dose compared to fluticasone propionate, (ii) a higher fraction of the drug deposited in the lungs, (iii) a lower permeability of budesonide resulting in a higher residence time of dissolved drug, as well as (iv) a faster dissolution, which leads to more dissolved drug in the lining fluids.

Besides drug- and formulation-specific parameters, the sensitivity analysis also provides insights into the most impactful physiological parameters, namely tissue volume, pulmonary perfusion as well as mucociliary clearance. This would mean that patients with a higher local perfusion would have a smaller pulmonary selectivity after oral inhalation of fluticasone propionate. To our knowledge, these physiological parameters are not available on an individual (patient) level, so that individual PK predictions are currently not possible with the here-presented model. Furthermore, it should be recognized that oral drug inhalation is characterized by high inter-occasional variability [72, 73], potentially being a result of varying inhalation characteristics such as the inhalation flow [74, 75], the inhaled volume (see S6 Fig) or also the actuation of the inhalation device [72]. Therefore, while we consider the mechanistic PDE modeling framework a good tool for optimizing drug or formulation characteristics, currently we do not consider it meaningful for performing individual PK predictions.

Even though this sensitivity analysis provides good insights into potential optimization parameters, it has to be recognized that varying a single input parameter at a time might not always be realistic. For example, a higher lipophilicity would result in slower dissolution kinetics, higher permeability, and higher tissue affinity. Therefore, as an extension of the here presented sensitivity analysis, a multi-parameter investigation might be meaningful during drug optimization. Alternatively, the model-based evaluation allows comparing completely different drugs in a drug optimization program to select the best drug candidate. However, here we evaluated the impact of the input parameters on the exposure in the conducting airways. These exposure metrics only represent surrogate parameters and have to be carefully selected based on the mode of action and the target location, i.e., for a target that would be located in the alveolar space other exposure metrics should be considered relevant for a sensitivity analysis.

In addition to identifying optimization parameters or potential reasons for inter-individual variability, this sensitivity analysis allows to identify the important physiological model parameters, which have to be understood to adequately predict the PK after oral inhalation. *Vice versa*, not knowing the exact values of less impactful (physiological) parameters is less critical to predict the drug exposure in human. The most impactful physiological parameters were tissue volume, perfusion, and mucociliary clearance. Less important physiological parameters were, for example, fluid volume or surface area. An additional highly uncertain parameter was the more central deposition pattern for asthmatic patients (which was corrected with an empirical correction factor). Therefore, to improve the PK predictions for patients, it would be valuable to generate and implement quantitative lung imaging data in patients [76]. As discussed before, the impactful parameters, including the deposition patterns, would also have to be understood on an individual level to ultimately predict individual (pulmonary) PK profiles. Another important uncertainty was the dissolution rate constant in the mucus. To our knowledge, no head-to-head comparison is available for *in vivo* relevant dissolution assays for both dissolution in the mucus and the alveolar lining fluids. This was why we had to make an assumption, namely a fivefold slower dissolution in the conducting airways compared to the alveolar lining fluid. The reason for these adapted dissolution rate constants is that the epithelial lining fluid in the conducting airways –the mucus– contains a lower concentration of surfactants, which facilitate dissolution [77], compared to the alveolar lining fluid. In addition, the upper layer of the mucus is characterized by a higher viscosity [78], which can also lead to a slower dissolution in comparison to the alveolar space. However, even though this assumption described the data well, it should be verified with *in vitro* dissolution experiments. In contrast, other uncertain (physiological) input parameters, such as the volume of the lung lining fluids, were not impactful and therefore could be considered less critical.

The previously mentioned data-based limitations also represent the main opportunities to improve the mechanistic PK model. First, it would significantly improve the applicability of the PK model framework if an adequate pulmonary deposition model for asthmatics could also be implemented (and later also for e.g., idiopathic pulmonary fibrosis). Furthermore, a more mechanistic representation of tissue distribution (e.g., separating extra- vs. intracellular concentrations) might increase the predictive power for drugs with a high pulmonary tissue binding. Adapting the model to clinical PK data (e.g., by estimating parameters) might improve the description of clinical data, but this would normally not be feasible during drug optimization. Therefore, no pulmonary PK parameters were estimated in this work.

In conclusion, a PDE-based fully mechanistic pulmonary PK model was developed to perform model-based predictions of the pulmonary and systemic pharmacokinetics of orally inhaled drugs based on *in vitro* formulation-specific, drug-specific, as well as physiological data. To our knowledge, this model is the first fully mechanistic and systematically evaluated pulmonary PK model. We also have shown that due to a large inter-study variability, model evaluation based on single (clinical) studies should be considered cautiously. This evaluated PK framework was applied to provide unique insights into optimization criteria for orally inhaled drugs by applying a model-based sensitivity analysis. It also provided insights which uncertainties of the modeling framework can still be improved. Overall, our analysis demonstrated that the model-based framework offers the potential to increase the quantitative understanding about inhaled drugs and ultimately, the model-based approach is applicable for optimizing drugs and formulations for inhalation therapy.

## Supporting information

S1 Appendix Model development and evaluation details. Derivations of model components from first principles, a description of the numerical resolution method, model evaluation against nonclinical data and the strategy for deposition adaptation for asthmatic patients.
(PDF)

S1 Fig Sensitivity analysis for budesonide. Additional sensitivity analyses for budesonide.
(PDF)

S2 Fig Deposition patterns. Deposition patterns in healthy volunteers and asthmatic patients for fluticasone propionate (Diskus) and budesonide (Turbohaler).
(PDF)

S3 Fig Concentration in lung lining fluids. Predicted time- and location-resolved lung lining fluid concentrations of fluticasone propionate (Diskus, 250 *μ*g dose) and budesonide (Turbohaler, 800 *μ*g dose).
(PDF)

S4 Fig Goodness-of-fit for lung retention data. Observed vs predicted pulmonary retention of inhaled gold and polystyrene (PSL) particles (see also Fig 3 in the main text).
(PDF)

S5 Fig Goodness-of-fit for pharmacokinetic data in healthy volunteers. Observed vs predicted dose-normalized plasma concentrations of fluticasone propionate (left) and budesonide (right), see also Fig 4 in the main text.
(PDF)

S6 Fig Sensitivity analysis—Pulmonary retention profiles for varying inhalation volumes of 5 *μ*m particles. Solid lines: predictions for different “bolus” inhalations ranging from 100–300 mL. Data points: raw data from [26], based on a “bolus depth” of up to 135 mL. Since the bolus enters the mouth-throat at a later stage in the experimental setup by [26] compared to natural tidal breathing which is predicted with the MPPD model, the tidal volume (here “inhalation volume”) is slightly larger than the “bolus depth”, with realistic inhalation volumes of ≈ 150–200 mL. A broader range of inhalation volumes is shown to illustrate the large impact of different inhalation characteristics.
(PDF)

S1 File MATLAB implementation. MATLAB scripts used for solving the PDE model and for evaluating it against clinical and experimental data.
(ZIP)

S1 Table Summary of pharmacokinetic studies. All identified clinical studies on fluticasone propionate and budesonide, including their study design and reported pharmacokinetic parameters.
(PDF)
