# Supplementary material for: A mechanistic framework for a priori pharmacokinetic predictions of orally inhaled drugs
Source: PLoS Comput Biol. 2020 Dec 15;16(12):e1008466. doi: 10.1371/journal.pcbi.1008466 (PMC7771877; doi:10.1371/journal.pcbi.1008466)
Supplement: S1 Appendix — Derivations of model components from first principles, a description of the numerical resolution method, model evaluation against nonclinical data and the strategy for deposition adaptation for asthmatic patients. (PDF) [file pcbi.1008466.s001.pdf]

A mechanistic framework for *a priori* pharmacokinetic predictions of orally inhaled drugs

S1 Appendix

Niklas Hartung, Jens Borghardt

Contents

1 Derivation of the PDE model **2**
1.1 Derivation of the dissolution model . . . 2
1.2 Derivation of the mucociliary clearance model . . . 2
1.3 Individual and population states . . . 3
1.4 Derivation of physiologically-structured population models (PSPMs) . . . 3
1.4.1 Conducting airways . . . 3
1.4.2 Alveolar space . . . 4
1.5 Mass balances . . . 4

2 Numerical resolution of the PDE model **6**
2.1 Notation . . . 6
2.2 Upwind discretization of physiologically-structured population equations . . . 7
2.3 Implicit discretization of linear processes . . . 7
2.4 Mass conservation of PDE discretisation . . . 9
2.5 Projections onto the computational grid . . . 9
2.5.1 Deposition patterns . . . 9
2.5.2 Per-generation parameters . . . 10

3 Additional model evaluations **10**
3.1 Evaluation of dissolution model against in vitro data . . . 10
3.2 Representation of lining fluid height in conducting airways based on literature data . . . 11
3.3 Evaluation of Usmani data . . . 12

4 Simulation of pulmonary deposition patterns **13**
4.1 Settings in MPPD software . . . 13
4.2 Adaptation of deposition patterns for asthmatic patients . . . 14

5 Generation of in-house data **16**
5.1 In vitro solubility determination in surfactant containing medium . . . 16
5.2 Blood to plasma ratio determination . . . 16

1 Derivation of the PDE model

1.1 Derivation of the dissolution model

The Noyes-Whitney equation [1] describes the dissolution flux $\frac{\mathrm{d}W}{\mathrm{dt}}$ in terms of properties of the dissolving particles and the dissolution medium,

$$\frac{\mathrm{d}W}{\mathrm{dt}} = -\frac{D \cdot \mathrm{SA}}{h}(C_\mathrm{s} - C_\mathrm{flu}), \tag{1}$$

where D is particle diffusivity, SA particle surface area, h height of the diffusion layer, C_s particle solubility and C_flu concentration of dissolved substance in the medium.

Through geometric assumptions on particles, this equation can be turned into a differential equation describing the change of volume of a dissolving particle. We assume particles to be spherical in shape, with radius r, surface area $\mathrm{SA} = 4\pi r^2$, volume $s = \cdot\frac{4}{3}\pi r^3$ and mass $W = \rho s$. Furthermore, as suggested previously [2], we assume the height of the diffusion layer to equate particle radius, $h \approx r$.

Since parametrizing the model in terms of radius r leads to a singularity of the dissolution model when $r \searrow 0$, in contrast to [2] we choose particle volume $s = \cdot\frac{4}{3}\pi r^3$ as a size descriptor instead of particle radius.

Differentiating the particle mass equation,

$$\frac{\mathrm{d}W}{\mathrm{dt}}(t) = \rho\frac{\mathrm{d}s}{\mathrm{dt}}(t), \tag{2}$$

and equating Eqs. (1) and (2) yields

$$\frac{\mathrm{d}s}{\mathrm{dt}}(t) = -\frac{D \cdot 4\pi r(t)^2}{\rho\, r(t)}(C_\mathrm{s} - C_\mathrm{flu}) = -\frac{D \cdot 4\pi r(t)}{\rho}(C_\mathrm{s} - C_\mathrm{flu}) = -\frac{D \cdot 4\pi\left(\frac{s(t)}{\frac{4}{3}\pi}\right)^{1/3}}{\rho}(C_\mathrm{s} - C_\mathrm{flu}).$$

We opt to parametrize the dissolution model in terms of maximum dissolution rate $k_\mathrm{diss} = D \cdot C_s$ rather than diffusivity D, since dissolution rate can be identified more directly from in vitro experiments (see Section Evaluation of dissolution model against in vitro data). The resulting dissolution model reads

$$d(s, C_\mathrm{flu}) = \frac{4\pi\, k_\mathrm{diss}}{(\frac{4}{3}\pi)^{1/3}\,\rho} \cdot \left(1 - \frac{C_\mathrm{flu}}{C_s}\right) \cdot s^{1/3}, \qquad \frac{\mathrm{d}s}{\mathrm{dt}}(t) = -d\big(s(t), C_\mathrm{flu}\big).$$

The concentration of dissolved substance, C_flu, also changes during dissolution. These processes are coupled in the PDE model described below.

1.2 Derivation of the mucociliary clearance model

As explained in the main text, a continuous representation of airway radius $r(x)$ depending on location x within the conducting airways is derived by interpolation. Using the Hofmann/Sturm model

$$v = 0.12553\frac{\mathrm{cm}}{\mathrm{min}} \cdot \left(\frac{d}{1\ \mathrm{cm}}\right)^{2.808},$$

we obtain a location-dependent mucociliary clearance model for a particle at location $x(t)$ at time t:

$$\frac{\mathrm{d}x}{\mathrm{dt}}(t) = -\lambda_\mathrm{mc}\big(x(t)\big) = 0.12553\frac{\mathrm{cm}}{\mathrm{min}} \cdot \left[2r^\mathrm{br}\left(\frac{x(t)}{1\ \mathrm{cm}}\right)\right]^{2.808} = -0.8791\frac{\mathrm{cm}}{\mathrm{min}} \cdot r^\mathrm{br}\left(\frac{x(t)}{1\ \mathrm{cm}}\right)^{2.808}.$$

1.3 Individual and population states

Physiologically-structured models describe the time evolution of a set of individuals/particles, each exhaustively described by a vector of characteristics called *state*, denoted z, and which changes over time. The time evolution of the state of any individual is assumed to be governed by a law G, i.e.

$$\frac{\mathrm{d}z}{\mathrm{dt}}(t) = G(t, z(t)), \quad z(0) = z_0.$$

Assuming that a population consists of a large number of individuals, it is natural not to describe each single individual but rather the time evolution of a density $\rho(t, z)$ of individuals over the state space. In this representation, the total number of particles is given by

$$N(t) = \int \rho(t, z)\mathrm{d}z,$$

and the number of particles within a particular subregion ω of the state space is given by

$$N_\omega(t) = \int_\omega \rho(t, z)\mathrm{d}z.$$

For such a domain ω, we set $\omega(t) = \{z(t) : z_0 \in \omega\}$. Assuming that the number of individuals is conserved in the state space, we obtain

$$\frac{\mathrm{d}}{\mathrm{dt}} N_{\omega(t)}(t) \equiv 0$$

as long as $\omega(t)$ does not touch the state space boundary. From this expression, a so-called continuity equation can be derived (see [3]):

$$\partial_t \rho(t, z) + \mathrm{div}_z \big[G(t, z)\rho(t, z)\big] = 0. \tag{3}$$

1.4 Derivation of physiologically-structured population models (PSPMs)

In our application context, the population consists of inhaled undissolved drug particles of different sizes, deposited at different locations within the conducting airways or within the alveolar space. The number of particles can only change if particles are (i) cleared to the GI tract by the mucociliary elevator (mucociliary clearance beyond the trachea, $x(t) = 0$) or (ii) completely dissolved ($s(t) = 0$).

1.4.1 Conducting airways

The particle state $z = (x, s) \in [0, x_{\mathrm{TB}}] \times [0, s_{\max}]$ can change by mucociliary clearance or dissolution (illustrated in Fig 1):

$$\begin{pmatrix} \frac{\mathrm{d}x}{\mathrm{dt}}(t) \\ \frac{\mathrm{d}s}{\mathrm{dt}}(t) \end{pmatrix} = \underbrace{\begin{pmatrix} -\lambda_{\mathrm{mcc}}(x(t)) \\ -d(s(t), C^{\mathrm{br}}_{\mathrm{flu}}(x(t), t)) \end{pmatrix}}_{=:G^{\mathrm{br}}(t, x(t), s(t))},$$

and Eq. (3) yields the location- and size-structured bronchial PSPM

$$\partial_t \rho^{\mathrm{br}}(t, x, s) - \partial_x \Big[\lambda_{\mathrm{mcc}}(x)\rho^{\mathrm{br}}(t, x, s)\Big] - \partial_s \Big[d\big(s, C^{\mathrm{br}}_{\mathrm{flu}}(t, x)\big)\rho^{\mathrm{br}}(t, x, s)\Big] = 0. \tag{4}$$

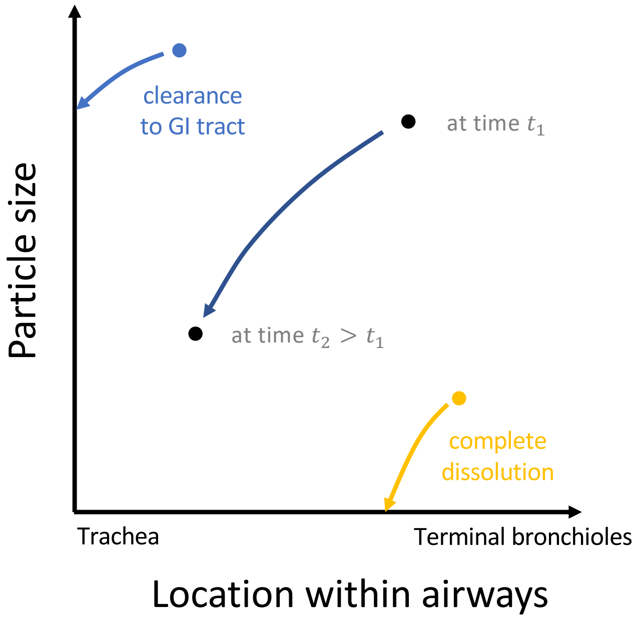

Figure 1: Phase plane representation of a drug particle in the conducting airways. Each particle is characterized by its location and size. Over time, particles move within this two-coordinate system until they are either cleared to the GI tract or completely dissolved.

1.4.2 Alveolar space

Since mucociliary clearance is not present in the alveolar space, the particle state $z = s \in [0, s_{\max}]$ can change by dissolution only:

$$\frac{\mathrm{d}s}{\mathrm{d}t}(t) = \underbrace{-d(s(t), C_{\mathrm{flu}}^{\mathrm{alv}}(t))}_{=:G^{\mathrm{alv}}(t,s(t))},$$

and Eq. (3) yields the size-structured alveolar PSPM

$$\partial_t \rho^{\mathrm{alv}}(t,s) - \partial_s \Big[d\big(s, C_{\mathrm{flu}}^{\mathrm{alv}}(t)\big) \rho^{\mathrm{alv}}(t,s) \Big] = 0.$$

1.5 Mass balances

When coupling the PSPM models to equations for dissolved drug in lining fluids, the number of molecules (not particles) have to be conserved during dissolution and mucociliary clearance. This model feature is ensured by deriving dissolution and mucociliary clearance rates directly from the PSPMs, which is shown in the following. The number of undissolved molecules in the conducting airways / the alveolar space are given by

$$A_{\mathrm{sol}}^{\mathrm{br}}(t) = \int_0^{x_{\mathrm{TB}}} \int_0^{s_{\max}} s \rho^{\mathrm{br}}(t,x,s) \mathrm{d}x \mathrm{d}s, \qquad A_{\mathrm{sol}}^{\mathrm{alv}}(t) = \int_0^{s_{\max}} s \rho^{\mathrm{alv}}(t,s) \mathrm{d}s.$$

We illustrate the derivation for the conducting airways, using integration by parts at step $(*)$:

$$
\begin{aligned}
\frac{\mathrm{d}A_{\mathrm{sol}}^{\mathrm{br}}}{\mathrm{dt}}(t) &= \int_0^{x_{\mathrm{TB}}}\int_0^{s_{\max}} s\partial_t\rho^{\mathrm{br}}(t,x,s)\mathrm{d}x\mathrm{d}s \\
&\overset{(4)}{=} -\int_0^{x_{\mathrm{TB}}}\int_0^{s_{\max}} s\Big(-\partial_x[\lambda_{\mathrm{mcc}}(x)\rho^{\mathrm{br}}(t,x,s)] - \partial_s[d(s,C_{\mathrm{flu}}^{\mathrm{br}}(t,x))\rho^{\mathrm{br}}(t,x,s)]\Big)\mathrm{d}x\mathrm{d}s \\
&\overset{(*)}{=} \int_0^{s_{\max}} s\big(\lambda_{\mathrm{mcc}}(x_{\mathrm{TB}})\underbrace{\rho^{\mathrm{br}}(t,\,x_{\mathrm{TB}},\,s)}_{=0 \text{ (no inflow)}} - \lambda_{\mathrm{mcc}}(0)\rho^{\mathrm{br}}(t,0,s)\big)\mathrm{d}s \\
&\quad -\int_0^{x_{\mathrm{TB}}}\int_0^{s_{\max}} d\big(s,C_{\mathrm{flu}}^{\mathrm{br}}(t,x)\big)\rho^{\mathrm{br}}(t,x,s)\mathrm{d}x\mathrm{d}s + \int_0^{x_{\mathrm{TB}}} s_{\max}d\big(s_{\max},C_{\mathrm{flu}}^{\mathrm{br}}(t,x)\big)\underbrace{\rho^{\mathrm{br}}(t,x,s_{\max})}_{=0 \text{ (no inflow)}}\mathrm{d}x \\
&= \underbrace{-\int_0^{s_{\max}} s\,\lambda_{\mathrm{mcc}}(0)\rho^{\mathrm{br}}(t,0,s)\mathrm{d}s}_{\text{cleared by mucociliary elevator}} \underbrace{-\int_0^{x_{\mathrm{TB}}}\int_0^{s_{\max}} d\big(s,C_{\mathrm{flu}}^{\mathrm{br}}(t,x)\big)\rho^{\mathrm{br}}(t,x,s)\mathrm{d}x\mathrm{d}s}_{\text{dissolved into lining fluids}}
\end{aligned}
$$

A similar but simplified reasoning applies to the alveolar space, where only dissolution, not mucociliary clearance, needs to be considered.

2 Numerical resolution of the PDE model

2.1 Notation

We consider a uniform time discretization step $\Delta t > 0$, a location discretization

$$0 = x_{1/2} < ... < x_{K+1/2} = x_{\text{TB}}$$

and a size discretisation

$$0 = s_{1/2} < ... < s_{L+1/2} = s_{\max}$$

These discretization points are understood as vertices of mesh elements $(k, l) = [x_{k-1/2}, x_{k+1/2}] \times [s_{l-1/2}, s_{l+1/2}]$ within which unknowns (approximations of ρ^{br}, $C^{\text{br}}_{\text{flu}}$, etc.) are defined; they appear in the discretization of the location- and size-structured model in the conducting airways. The same size grid is also used when discretizing the size-structured model in the alveolar space. Furthermore, we define the center (x_k, s_l) of mesh element (k, l) from the above discretization points,

$$x_k := \frac{x_{k-1/2} + x_{k+1/2}}{2}, \qquad k \in \{1, .., K\},$$
$$s_l := \frac{s_{l-1/2} + s_{l+1/2}}{2}, \qquad l \in \{1, .., L\}.$$

We use the following notation:

- $\Delta x_k := x_{k+1/2} - x_{k-1/2}$ (location length of mesh element $(k, \cdot)$)
- $\Delta s_l := s_{l+1/2} - s_{l-1/2}$ (size length of mesh element $(\cdot, l)$); we also define $\Delta s_{l+1/2} := s_{l+1} - s_l$ (this expression will appear later during computations)
- Abbreviations for location-structured physiology in conducting airways: $\lambda_k := \lambda_{\text{mc}}(x_k)$, $r^{\text{br}}_k := r^{\text{br}}(x_k)$, $q^{\text{br}}_k := q^{\text{br}}(x_k)$, $a^{\text{br}}_{\text{flu},k} := a^{\text{br}}_{\text{flu}}(x_k)$, $a^{\text{br}}_{\text{tis},k} := a^{\text{br}}_{\text{tis}}(x_k)$
- $\rho^{\text{br},n}_{k,l}$ as the numerical approximation of $\rho^{\text{br}}(t_n, x_k, s_l)$
- $\rho^{\text{alv},n}_{l}$ as the numerical approximation of $\rho^{\text{alv}}(t_n, s_l)$
- $C^{\text{br},n}_{\text{flu},k}$ as the numerical approximation of $C^{\text{br}}_{\text{flu}}(t_n, x_k)$
- $C^{\text{br},n}_{\text{tis},k}$ as the numerical approximation of $C^{\text{br}}_{\text{tis}}(t_n, x_k)$
- $C^{\text{alv},n}_{\text{flu}}$ as the numerical approximation of $C^{\text{alv}}_{\text{flu}}(t_n)$
- $C^{\text{alv},n}_{\text{tis}}$ as the numerical approximation of $C^{\text{alv}}_{\text{tis}}(t_n)$
- $A^{\text{y},n}_{x}$ as the numerical approximation of $A^{\text{y}}_{x}(t_n)$ (total amount of drug in a certain state; one of $A^{\text{br}}_{\text{sol}}$, $A^{\text{br}}_{\text{flu}}$, $A^{\text{br}}_{\text{tis}}$, $A^{\text{alv}}_{\text{sol}}$, $A^{\text{alv}}_{\text{flu}}$, $A^{\text{alv}}_{\text{tis}}$, $A^{\text{clear}}_{\text{mcc}}$, $A^{\text{clear}}_{\text{sys}}$, $A^{\text{sys}}_{\text{tot}}$), with 'sol' meaning 'solid', i.e. undissolved.

2.2 Upwind discretization of physiologically-structured population equations

Upwind discretizations, i.e. non-centered finite difference approximations depending on the flow direction, are well tailored to PSPMs, resulting in stable discretizations as long as the timestep Δt is small enough (called a CFL condition).

The upwind discretization of the conducting airway PSPM

$$\partial_t \rho^{\mathrm{br}}(t,x,s) - \partial_x\big[\lambda_{\mathrm{mc}}(x)\rho^{\mathrm{br}}(t,x,s)\big] - \partial_s\big[d(s, C^{\mathrm{br}}_{\mathrm{flu}}(t,x))\rho^{\mathrm{br}}(t,x,s)\big] = 0$$

is given by

$$\frac{\rho^{\mathrm{br},n+1}_{k,l} - \rho^{\mathrm{br},n}_{k,l}}{\Delta t} - \frac{\lambda_{k+1/2}\rho^{\mathrm{br},n}_{k+1,l} - \lambda_{k-1/2}\rho^{\mathrm{br},n}_{k,l}}{\Delta x_k} - \frac{d(s_{l+1/2}, C^{\mathrm{br},n}_{\mathrm{flu},k})\rho^{\mathrm{br},n}_{k,l+1} - d(s_{l-1/2}, C^{\mathrm{br},n}_{\mathrm{flu},k})\rho^{\mathrm{br},n}_{k,l}}{\Delta s_l} = 0,$$

for $n \in \{1,...,N\}, k \in \{1,...,K\}, l \in \{1,...,L\}$ (with $\rho^{\mathrm{br},n}_{K+1,l} = \rho^{\mathrm{br},n}_{k,L+1} = 0$, i.e. no inflow condition). Similarly the upwind discretization of the alveolar PSPM

$$\partial \rho^{\mathrm{alv}}(t,s) - \partial_s\big[d(s, C^{\mathrm{alv}}_{\mathrm{flu}}(t))\rho^{\mathrm{alv}}(t,s)\big] = 0$$

is given by

$$\frac{\rho^{\mathrm{alv,n+1}}_{l} - \rho^{\mathrm{alv},n}_{l}}{\Delta t} - \frac{d(s_{l+1/2}, C^{\mathrm{alv},n}_{\mathrm{flu}})\rho^{\mathrm{alv},n}_{l+1} - d(s_{l-1/2}, C^{\mathrm{alv},n}_{\mathrm{flu}})\rho^{\mathrm{alv},n}_{l}}{\Delta s_l} = 0.$$

Within this framework, the number of undissolved drug molecules is approximated by

$$A^{\mathrm{br},n}_{\mathrm{sol,k}} := \sum_{l=1}^{L} \Delta s_l s_l \rho^{\mathrm{br},n}_{k,l} \qquad \text{(location } k \text{ in conducting airways)},$$

$$A^{\mathrm{alv},n}_{\mathrm{sol}} := \sum_{l=1}^{L} \Delta s_l s_l \rho^{\mathrm{alv},n}_{l} \qquad \text{(alveolar space)}.$$

2.3 Implicit discretization of linear processes

Recognizing that all processes except for dissolution and mucociliary clearance are linear, we propose an implicit discretization to ensure unconditional stability of these other processes, too. The numerical scheme is formulated in terms of local amounts (in bronchial/alveolar fluid/tissue) rather than concentrations. To this end, we define

$$V^{\mathrm{br}}_{\mathrm{flu},k} := \Delta x_k\, a^{\mathrm{br}}_{\mathrm{flu},k} \qquad \text{(lining fluid volume at } k\text{-th location grid cell)}$$

$$V^{\mathrm{br}}_{\mathrm{tis},k} := \Delta x_k\, a^{\mathrm{br}}_{\mathrm{tis},k} \qquad \text{(tissue volume at } k\text{-th location grid cell)}$$

and obtain the amounts

$$A^{\mathrm{br},n}_{\mathrm{flu},k} := C^{\mathrm{br},n}_{\mathrm{flu},k} V^{\mathrm{br}}_{\mathrm{flu},k}, \qquad A^{\mathrm{br},n}_{\mathrm{tis},k} := C^{\mathrm{br},n}_{\mathrm{tis},k} V^{\mathrm{br}}_{\mathrm{tis},k} \qquad \text{(conducting airways)},$$

$$A^{\mathrm{alv},n}_{\mathrm{flu}} := C^{\mathrm{alv},n}_{\mathrm{flu}} V^{\mathrm{alv}}_{\mathrm{flu}}, \qquad A^{\mathrm{alv},n}_{\mathrm{tis}} := C^{\mathrm{alv},n}_{\mathrm{tis}} V^{\mathrm{alv}}_{\mathrm{tis}} \qquad \text{(alveolar space)}.$$

Furthermore, it will be useful to define

$$\mathrm{PS}^{\mathrm{br}}_{\mathrm{k}} := \Delta x_k\, 2\pi r^{\mathrm{br}}_k\, P_{\mathrm{app}} \qquad \text{(permeability-surface area product at } k\text{-th location grid cell)}$$

$$Q^{\mathrm{br}}_k := \Delta x_k\, q^{\mathrm{br}}_k \qquad \text{(perfusion of } k\text{-th location grid cell)}.$$

To arrive at a numerical scheme formulated on the computational grid, integrals are discretized as follows:

$$\int_0^{\mathrm{s_{max}}} f(s)\mathrm{d}s \quad \Rightarrow \quad \sum_{l=1}^{L} \Delta s_l f(s_l), \qquad \int_0^{\mathrm{x_{TB}}} f(x)\mathrm{d}x \quad \Rightarrow \quad \sum_{k=1}^{K} \Delta x_k f(x_k)$$

Bronchial kinetics

$$\frac{A_{\mathrm{flu},k}^{\mathrm{br},n+1} - A_{\mathrm{flu},k}^{\mathrm{br},n}}{\Delta t} = \underbrace{\Delta x_k \sum_{l=2}^{L} \Delta s_{l-1/2} d(s_{l-1/2}, C_{\mathrm{flu},k}^{\mathrm{br},n}) \rho_{k,l}^{\mathrm{br},n}}_{\text{dissolved (see section below)}} - \mathrm{PS}_{\mathrm{k}}^{\mathrm{br}} \left(\frac{A_{\mathrm{flu},k}^{\mathrm{br},n+1}}{V_{\mathrm{flu},k}^{\mathrm{br}}} - \frac{A_{\mathrm{tis},k}^{\mathrm{br},n+1}}{V_{\mathrm{tis},k}^{\mathrm{br}} K_{pl,u}} \right)$$

$$\frac{A_{\mathrm{tis},k}^{\mathrm{br},n+1} - A_{\mathrm{tis},k}^{\mathrm{br},n}}{\Delta t} = \mathrm{PS}_{\mathrm{k}}^{\mathrm{br}} \left(\frac{A_{\mathrm{flu},k}^{\mathrm{br},n+1}}{V_{\mathrm{flu},k}^{\mathrm{br}}} - \frac{A_{\mathrm{tis},k}^{\mathrm{br},n+1}}{V_{\mathrm{tis},k}^{\mathrm{br}} K_{pl,u}} \right) - Q_k^{\mathrm{br}} \left(\frac{A_{\mathrm{tis},k}^{\mathrm{br},n+1}}{V_{\mathrm{tis},k}^{\mathrm{br}}} \frac{R}{K_{pl}} - \frac{A_{\mathrm{ctr}}^{\mathrm{sys},n+1}}{V_{\mathrm{ctr}}^{\mathrm{sys}}} \right)$$

Alveolar kinetics

$$\frac{A_{\mathrm{flu}}^{\mathrm{alv},n+1} - A_{\mathrm{flu}}^{\mathrm{alv},n}}{\Delta t} = \sum_{l=2}^{L} \Delta s_{l-1/2} d(s_{l-1/2}, C_{\mathrm{flu}}^{\mathrm{alv},n}) \rho_{l}^{\mathrm{alv},n} - \mathrm{PS}^{\mathrm{alv}} \left(\frac{A_{\mathrm{flu}}^{\mathrm{alv},n+1}}{V_{\mathrm{flu}}^{\mathrm{alv}}} - \frac{A_{\mathrm{tis}}^{\mathrm{alv},n+1}}{V_{\mathrm{tis}}^{\mathrm{alv}} K_{pl,u}} \right)$$

$$\frac{A_{\mathrm{tis}}^{\mathrm{alv},n+1} - A_{\mathrm{tis}}^{\mathrm{alv},n}}{\Delta t} = \mathrm{PS}^{\mathrm{alv}} \left(\frac{A_{\mathrm{flu}}^{\mathrm{alv},n+1}}{V_{\mathrm{flu}}^{\mathrm{alv}}} - \frac{A_{\mathrm{tis}}^{\mathrm{alv},n+1}}{V_{\mathrm{tis}}^{\mathrm{alv}} K_{pl,u}} \right) - Q^{\mathrm{alv}} \left(\frac{A_{\mathrm{tis}}^{\mathrm{alv},n+1}}{V_{\mathrm{tis}}^{\mathrm{alv}}} \frac{R}{K_{pl}} - \frac{A_{\mathrm{ctr}}^{\mathrm{sys},n+1}}{V_{\mathrm{ctr}}^{\mathrm{sys}}} \right)$$

Systemic kinetics

$$\begin{aligned}
\frac{A_{\mathrm{gut}}^{\mathrm{sys},n+1} - A_{\mathrm{gut}}^{\mathrm{sys},n}}{\Delta t} &= \qquad \underbrace{\sum_{l=1}^{L} \Delta s_l s_l \lambda_{1/2} \rho_{1,l}^{\mathrm{br},n}}_{\text{mucociliary clearance (see section below)}} \qquad -k_{01} A_{\mathrm{gut}}^{\mathrm{sys},n+1} \\
\frac{A_{\mathrm{ctr}}^{\mathrm{sys},n+1} - A_{\mathrm{ctr}}^{\mathrm{sys},n}}{\Delta t} &= F k_{01} A_{\mathrm{gut}}^{\mathrm{sys},n+1} - k_{12} A_{\mathrm{ctr}}^{\mathrm{sys},n+1} + k_{21} A_{\mathrm{per}}^{\mathrm{sys},n+1} \\
&\quad + Q^{\mathrm{alv}} \left(\frac{A_{\mathrm{tis}}^{\mathrm{alv},n+1}}{V_{\mathrm{tis}}^{\mathrm{alv}}} \frac{R}{K_{pl}} - \frac{A_{\mathrm{ctr}}^{\mathrm{sys},n+1}}{V_{\mathrm{ctr}}^{\mathrm{sys}}} \right) \\
&\quad + \sum_{k=1}^{K} Q_k^{\mathrm{br}} \left(\frac{A_{\mathrm{tis},k}^{\mathrm{br},n+1}}{V_{\mathrm{tis},k}^{\mathrm{br}}} \frac{R}{K_{pl}} - \frac{A_{\mathrm{ctr}}^{\mathrm{sys},n+1}}{V_{\mathrm{ctr}}^{\mathrm{sys}}} \right) \\
\frac{A_{\mathrm{per}}^{\mathrm{sys},n+1} - A_{\mathrm{per}}^{\mathrm{sys},n}}{\Delta t} &= k_{12} A_{\mathrm{ctr}}^{\mathrm{sys},n+1} - k_{21} A_{\mathrm{per}}^{\mathrm{sys},n+1} \\
\frac{A_{\mathrm{clear}}^{n+1} - A_{\mathrm{clear}}^{n}}{\Delta t} &= (1-F) k_{01} A_{\mathrm{gut}}^{\mathrm{sys},n+1} + k_{10} A_{\mathrm{ctr}}^{\mathrm{sys},n+1}
\end{aligned}$$

2.4 Mass conservation of PDE discretisation

The above terms are chosen such that the number of molecules is conserved, i.e., the total amount of drug in the body plus the amount excreted, given by

$$A_{\text{tot}}^n = \underbrace{A_{\text{sol}}^{\text{br},n} + \sum_{k=1}^{K}\left(A_{\text{flu,k}}^{\text{br},n} + A_{\text{tis,k}}^{\text{br},n}\right)}_{\text{in conducting airways}} + \underbrace{A_{\text{sol}}^{\text{alv},n} + A_{\text{flu}}^{\text{alv},n} + A_{\text{tis}}^{\text{alv},n}}_{\text{in alveolar space}} + \underbrace{A_{\text{ctr}}^{\text{sys},n} + A_{\text{per}}^{\text{sys},n} + A_{\text{gut}}^{\text{sys},n} + A_{\text{clear}}^{n}}_{\text{in GI tract, systemic circulation or excreted}},$$

remains constant for all n. Mass conservation during uptake from lining fluid to lung tissue can be seen directly from the rates in the equations: the same terms, e.g.

$$\text{PS}_{\text{k}}^{\text{br}}\left(\frac{A_{\text{flu},k}^{\text{br},n+1}}{V_{\text{flu},k}^{\text{br}}} - \frac{A_{\text{tis},k}^{\text{br},n+1}}{V_{\text{tis},k}^{\text{br}}\,K_{pl,u}}\right),$$

appear in both equations with opposing signs (for systemic uptake from conducting airway tissue, contributions at different locations are summed). Furthermore, using the upwind formulation, we can decompose the rate of change of the amount of undissolved drug:

$$\begin{aligned}
\frac{A_{\text{sol}}^{\text{br},n+1} - A_{\text{sol}}^{\text{br},n}}{\Delta t} &= \sum_{k=1}^{K}\sum_{l=1}^{L}\Delta x_k \Delta s_l\, s_l \frac{\rho_{k,l}^{\text{br},n+1} - \rho_{k,l}^{\text{br},n}}{\Delta t} \\
&= -\sum_{k=1}^{K}\sum_{l=1}^{L}\Delta x_k \Delta s_l\, s_l\left(-\frac{\lambda_{k+1/2}\rho_{k+1,l}^{\text{br},n} - \lambda_{k-1/2}\rho_{k,l}^{\text{br},n}}{\Delta x_k} - \frac{d(s_{l+1/2}, C_{\text{flu},k}^{\text{br},n})\rho_{k,l+1}^{\text{br},n} - d(s_{l-1/2}, C_{\text{flu},k}^{\text{br},n})\rho_{k,l}^{\text{br},n}}{\Delta s_l}\right) \\
&= +\sum_{k=1}^{K}\sum_{l=1}^{L}\Delta s_l\, s_l\left(\lambda_{k+1/2}\rho_{k+1,l}^{\text{br},n} - \lambda_{k-1/2}\rho_{k,l}^{\text{br},n}\right) \\
&\quad + \sum_{k=1}^{K}\sum_{l=1}^{L}\Delta x_k s_l\left(d(s_{l+1/2}, C_{\text{flu},k}^{\text{br},n})\rho_{k,l+1}^{\text{br},n} - d(s_{l-1/2}, C_{\text{flu},k}^{\text{br},n})\rho_{k,l}^{\text{br},n}\right) \\
&= -\underbrace{\sum_{l=1}^{L}\Delta s_l\, s_l \lambda_{1/2}\rho_{1,l}^{\text{br},n}}_{\text{mucociliary clearance}} - \sum_{k=1}^{K}\Delta x_k \underbrace{\sum_{l=2}^{L}\Delta s_{l-1/2} d(s_{l-1/2}, C_{\text{flu},k}^{\text{br},n})\rho_{k,l}^{\text{br},n}}_{\text{dissolution at location } k}
\end{aligned}$$

and noting that these two terms are matched in the equations for dissolved drug in the lining fluid and of cleared drug, we can conclude that mass is conserved during dissolution and mucociliary clearance. An analogous computation shows mass conservation during dissolution in the alveolar space. Mass balance was checked systematically during all simulations shown.

2.5 Projections onto the computational grid

Deposition patterns, as well as several parameters used in the PSPMs, are not resolved at the same scale as the computational grid. Therefore, a projection step is necessary prior to being able to integrate these quantities into the model.

2.5.1 Deposition patterns

Deposition data are given for each airway generation $g_1, ..., g_K$ and for a fixed set of reference particle sizes $S_1, ..., S_L$, resulting in a discrete deposition pattern $(D_{k,l})$. The dose should be conserved, equivalent to conservation of number of molecules, but not number of particles.

We proceeded as follows (see Fig. 2 for an illustration):

- We define a region S_k^ε around S_k, given by$S_k^\varepsilon = [S_k - \varepsilon, S_k + \varepsilon]$, with small ε such that all such regions are disjoint.
- From the discrete values $D_{k,l}$, we define a continuous function

$$D(x,s) = \sum_{k,l} \frac{1}{2\varepsilon|g_k|} \mathbb{1}_{\{x \in g_k, s \in [S_k - \varepsilon, S_k + \varepsilon]\}},$$

  such that $\int_{g_k \times S_k^\varepsilon} D(x,s)\mathrm{d}x\mathrm{d}s = D_{k,l}$.
- We define the initial condition on the computational grid by

$$\rho_{k,l}^0 = \frac{1}{\Delta x_k \, \Delta s_l} \int\limits_{C(k,l)} D(x,s)\mathrm{d}x\mathrm{d}s$$

  for grid cell $C(k,l) = \left[x_{k-\frac{1}{2}}, x_{k+\frac{1}{2}}\right] \times \left[s_{l-\frac{1}{2}}, s_{l+\frac{1}{2}}\right]$

2.5.2 Per-generation parameters

For a per-generation parameter (e.g., airway radius, blood flow, ...), generically denoted P, we construct a location-resolved representation using the previous construction only in the location coordinate, i.e.:

- From the discrete values P_k, we define a continuous function

$$P(x) = \sum_k \frac{1}{|g_k|} \mathbb{1}_{\{x \in g_k\}},$$

  such that $\int_{g_k} P(x)\mathrm{d}x = P_k$.
- We define the location-resolved representation on the computational grid by

$$p_k = \frac{1}{\Delta x_k} \int\limits_{x_{k-\frac{1}{2}}}^{x_{k+\frac{1}{2}}} P(x)\mathrm{d}x.$$

3 Additional model evaluations

3.1 Evaluation of dissolution model against in vitro data

We evaluated the dissolution model against *in vitro* data from a dissolution study [4], where the authors evaluated the dissolution kinetics of fluticasone propionate and budesonide particles with defined particle sizes (see Table 1).

Based on the in vitro data, we compared different dissolution models:

- a first-order dissolution model (estimated empirically; size-independent)
- an unsaturable dissolution model (formally corresponding to $C_s = +\infty$ in the dissolution model)
- saturable dissolution models with different solubilities

The results are shown in Fig. 3. A particle size-dependency is clearly visible, as well as a saturation effect. Among the different saturable dissolution models, the parametrization using in house data resulted in a qualitatively better description than the values reported in [4].

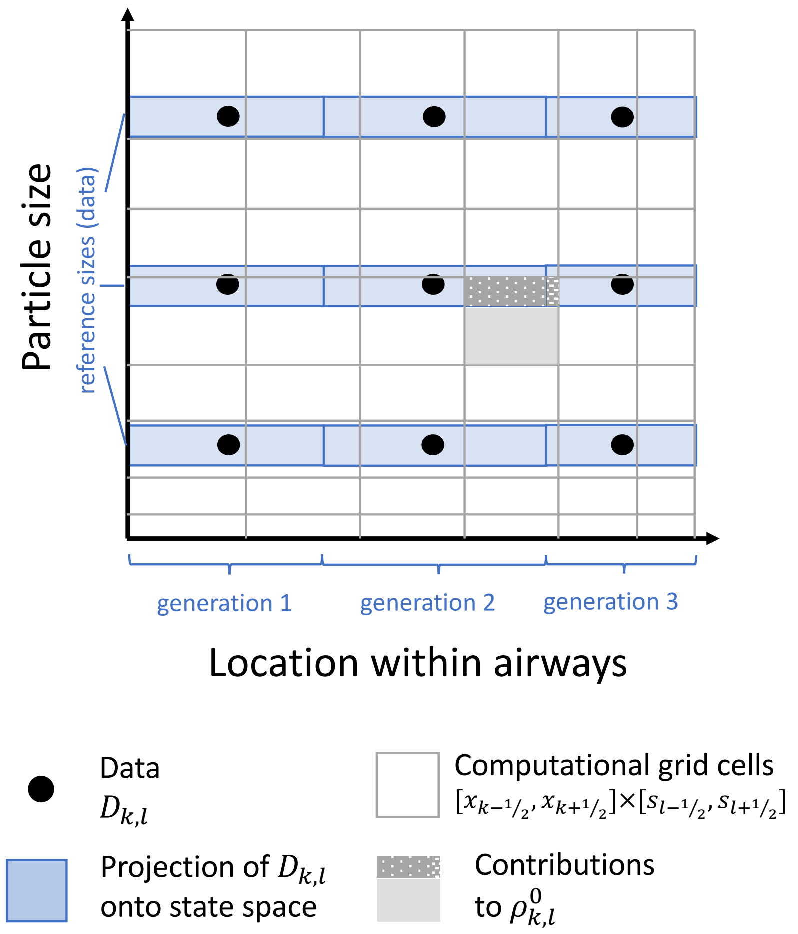

Figure 2: Resolution of data against computational grid. Deposited amounts of particles with a particular size and at a particular airway location (black dots) are first distributed evenly within the respective airway generation and a small size range (blue rectangles), yielding a continuous representation of deposition within state space. The numerical approximation to the location- and size-structured density is defined on an independent computational grid. Its initial value within a grid cell is the average of the values of the continuous representation. Contributing location-size regions to a particular grid cell are highlighted in gray.

3.2 Representation of lining fluid height in conducting airways based on literature data

Different values for the thickness of the lining fluid layer in the conducting airways have been reported. After reviewing the literature, we concluded that the linear relationship shown in Fig. 4 adequately described the current state of knowledge.

We decided not use literature values on total lung lining fluid volume since the reported values are not experimentally measured values but rather estimates based on height measurements and geometrical considerations. However, we note that the total lining fluid volume computed under the our geometrical

Substance	ACI stage	Cutoff size range	Aerodyn. diam.	Geometric diam.
Fluticasone propionate	4	2.1 – 3.3 μm	2.7 μm	3.2 μm
Fluticasone propionate	2	4.7 – 5.8 μm	5.25 μm	6.2 μm
Budesonide	4	2.1 – 3.3 μm	2.7 μm	2.4 μm

Table 1: Aerodynamic and geometric particle sizes corresponding to the experimental protocols of [4]. Particles within defined ranges of aerodynamic particle sizes were obtained from different stages of Anderson cascade impactors (ACI). For simulation of dissolution kinetics, we took the geometric diameter corresponding to the mean aerodynamic particle diameter within each impactor stage.

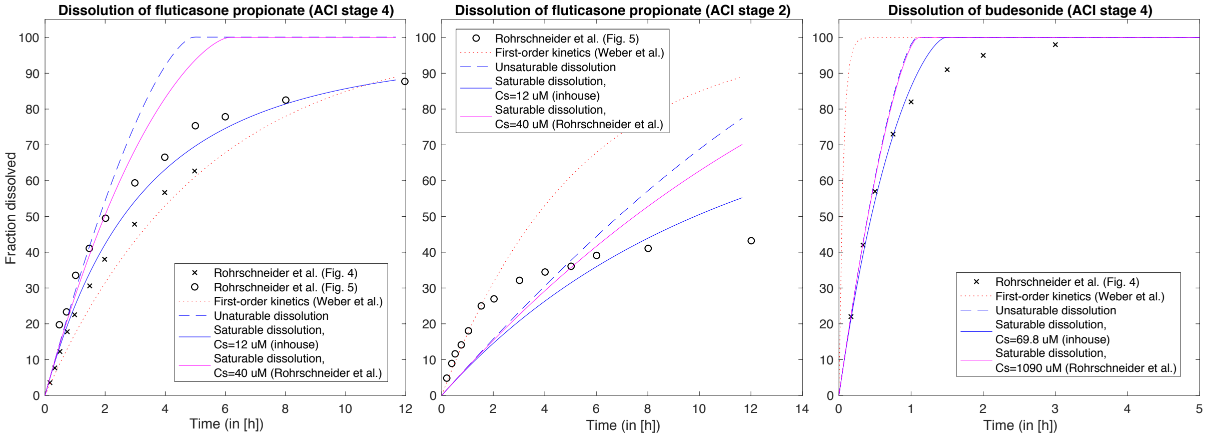

Figure 3: Comparison of dissolution models based on in vitro dissolution data.

assumptions ($\approx$ 1.2 mL) was smaller than the ones given in the literature (10–70 mL).

3.3 Evaluation of Usmani data

As stated in the main text, we could not reproduce the fluticasone propionate exposure indices reported by Usmani et al. [9] based on the provided study information. Here we provide full details for this statement. For the smallest particles of 1.5 μm diameter, Usmani et al. reported an $\mathrm{AUC}_{\text{0-12h}}$ of 923.28 pg $\cdot$ h/mL, i.e. in molar units 1.84 nM $\cdot$ h. Assuming 100 % lung uptake, no mucociliary clearance and a full systemic uptake within 12 h, and taking the literature value for fluticasone propionate clearance of 73 L/h [10], we obtain a very conservative upper bound of $\mathrm{AUC}_{\max} = \frac{\mathrm{Dose}}{\mathrm{CL}\cdot\mathrm{MW}} = 1.37$ nM $\cdot$ h.

A more realistic, albeit still conservative calculation and a simulation with the PDE model are shown in Table 2. In conclusion, the reported AUC value is approximately 2-4 times larger than what could be reasonably expected. Accordingly, $C_{\max}$ values are also much higher than predicted by the PDE model.

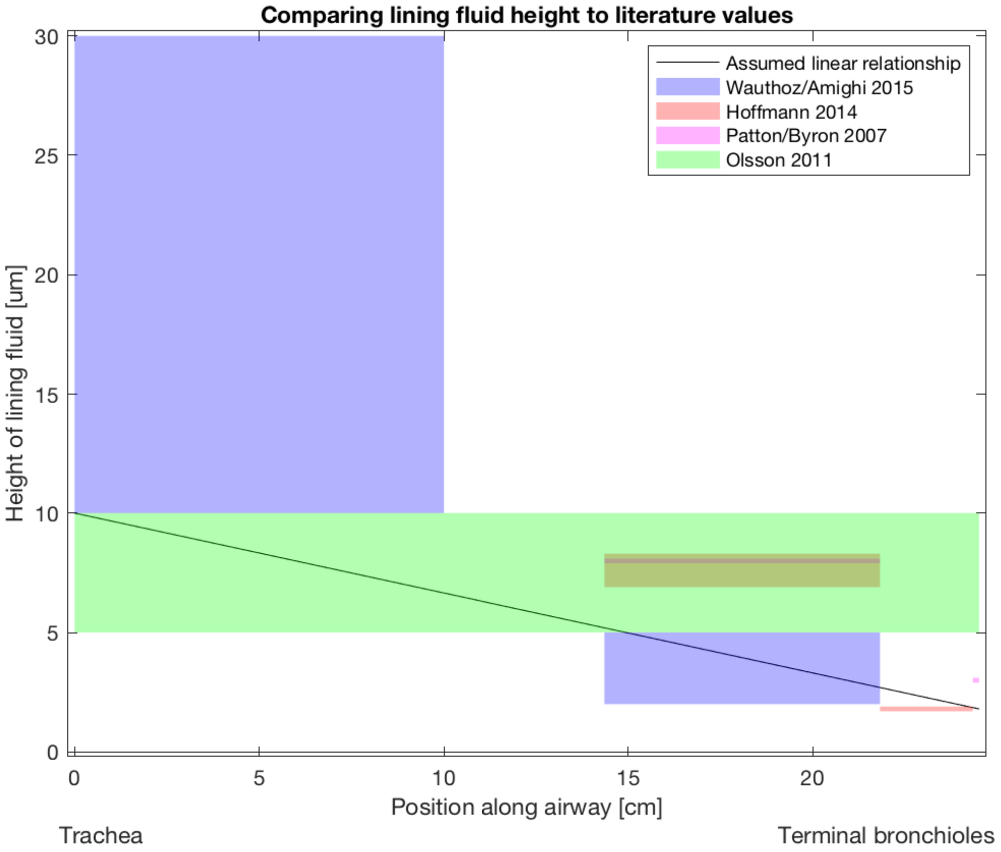

Figure 4: Model for height of location-resolved lining fluid (solid black line) compared to reported literature data [5, 6, 7, 8].

4 Simulation of pulmonary deposition patterns

4.1 Settings in MPPD software

In order to predict the pulmonary deposition patterns, the MPPD software v2.1 was used [11]. This software allows to predict the generation-dependent pulmonary deposition of inhaled particles, where generations 1-17 represent the conducting airways (generation 1 = trachea) and generations 18-25 the alveolar space. Three types of input data are required in the MPPD software: (1) airway morphometry, (2) particle properties, and (3) exposure condition, as outlined below. The MPPD software was only applied to simulate the deposition patterns but not used to investigate the clearance of particles from the lung.

Airway morphometry. For all predictions performed with the MPPD software, the airway morphometry was represented by the human "Yeh/Schum 5-Lobe" model [12]. The inhalation flow characteristics were assumed to be represented by uniform expansion of the lung so that consequently also the inhalation and exhalation flow were constant over time. The standard airway morphometry defined in the MPPD software was selected for all deposition pattern predictions, i.e. the default values for functional

Assumptions for AUC calculation			Calculated AUC
Lung dose	MCC	Timespan	(compared to reported AUC)
100%	no	$AUC_{0\text{-}\infty}$	26% lower than reported
56%	no	$AUC_{0\text{-}\infty}$	58% lower than reported
56%	yes	$AUC_{0\text{-}12h}$	74% lower than reported

Table 2: Comparison of calculated AUC vs. reported $AUC_{0\text{-}12h}$ for different assumptions. Even under the most conservative assumptions, AUC is considerably underestimated, which becomes more pronounced as the model gets more realistic.

residual capacity (3300 mL) and upper respiratory tract volume (50 mL) were used [13].

Particle properties. The inhaled particle properties were defined based on the information in the respective publications, or alternatively for the respective inhalation device (references are provided in Table 3). For all predictions, the density of the particles was set to 1 g/cm^3; and the particle diameter was defined as the mass median aerodynamic diameter, which is typically provided in literature. As described in the main manuscript, the difference between aerodynamic and geometric diameters was accounted for, such that the real surface area could be used as an input parameter to the dissolution model. The MPPD software was only used to predict pulmonary deposition patterns of monodisperse particles. To predict the deposition patterns for the monodisperse gold/polystyrene particles (Study I) and the inhaled monodisperse fluticasone propionate particles (Study II), this information was sufficient. Whenever pulmonary deposition patterns of a particle size distribution were required (Studies III/IV), these were generated in a two-step approach. First, all relevant monodisperse particles size bins of the particle size distribution were simulated as monodisperse particles with the MPPD software. In a second step, the complete deposition pattern was calculated by normalizing the deposited amount per particle size bin by the dose in this respective bin. The two additional options of the MPPD software, namely the "Nanoparticle Model" and "Inhalability Adjustment" were not applied to predict the deposition patterns.

Exposure conditions. The exposure scenario was set to constant exposure and the body orientation during the inhalation process was assumed "upright". Furthermore, for all predictions, it was assumed that the breathing scenario was represented by oral breathing, which is the typical inhalation route for drugs delivered to the lungs. Breathing frequency, tidal volume, inspiratory fraction as well as pause fraction were all defined based on the inhalation flow properties provided in the respective publications (see Table 3).

4.2 Adaptation of deposition patterns for asthmatic patients

Since the MPPD software predicts deposition patterns in healthy volunteers, it cannot directly be used to predict deposition patterns in asthmatic or COPD patients. In these patients, due to narrowed airways, deposition is more central in comparison to healthy volunteers. Whenever patients were considered in a study rather than healthy volunteers, deposition patterns had to be adapted adequately. To this end, the fraction of the inhaled dose deposited in any specific airway generation was increased by an adjustment factor such that the deposited fraction of the lung dose in the alveolar space was 2-fold lower than in healthy volunteers. This number was derived from published data on conducting airway to alveolar deposition ratios [17].

	**Study** I	**Study II**	**Study III**	**Study IV**
**Particle properties**				
Substance	gold / polystyrene	fluticasone propionate	fluticasone propionate	budesonide
Formulation type	monodisperse	monodisperse	polydisperse	polydisperse
Particle size(s)	5μm diameter	1.5 / 3 / 6 μm diameter	distribution based on [14]	distribution based on [14]
**Exposure scenario**				
Device	custom setup (see [15])	Inhalation chamber	Diskus®	Turbohaler®
Breathing frequency	6/min	5/min	6/min	6/min
Tidal volume	200 mL	2000 mL	2000 mL	2000 mL
Inhalation time	1 sec	4 sec	1.33 sec	1.33 sec
Exhalation time	1 sec	3 sec	2.67 sec	2.67 sec
Pause time	8 sec	5 sec	6 sec	6 sec
Inhalation flow	12 L/min	30 L/min	90 L/min	60 L/min
**Deposition pattern corrections**				
Lung dose	no correction	56.3% / 51% / 46.0%	14.5% based on [16]	35% based on [16]
Central/peripheral deposition ratio	no correction	central deposited fraction: 56.1% / 65.7% / 75.4%	2-fold lower alveolar deposition for asthma patients [17]	2-fold lower alveolar deposition for asthma patients [17]

Table 3: Study-specific input data to the MPPD software.

5 Generation of in-house data

5.1 In vitro solubility determination in surfactant containing medium

For *in vivo* relevant characterization of the drug solubility, the surfactant-containing medium Alveofact®, a commercially available product, was taken. Alveofact®contains phospholipids obtained from bovine lung (i.e., surfactants) and is available as dry powder ampoules ready for reconstitution. As reconstitution medium, a 0.1 mol/l sodium dihydrogenecarboante buffer with pH 7.4 was used. A suspension with 50 mg/ml Alveofact®was produced according to the information and instruction for use of the commercial product. At these concentrations, Alveofact®forms a micellar system. 1 mg of drug (either budesonide or fluticasone propionate) is suspended in 1 ml of this medium and shaken for 24 h at 37 °C. Afterwards, the suspension is filtered with a commercially available Whatman Mini-UniPrep syringeless filter containing a 0.45 μm filter membrane out of glass microfibers. As the micelles pass this membrane and as the concentration of phospholipids is too high to be directly injected in the HPLC system for analysis of the solubilized amount of drug, the micelles are destroyed by adding DMSO in a 1:1 ratio to the filtered micellar solution. The phospholipids can be separated by an additional 5 – 10 minutes centrifugation step. A small aliquot of the remaining solution is taken and injected into a HPLC system for quantitative analysis of the solubilized amount of drug.

5.2 Blood to plasma ratio determination

To determine the Blood:Plasma (BP) ratio, the respective amount of the drug (i.e., fluticasone propionate) was added to 490 μL human blood and to 490 μL plasma samples to obtain a drug concentration of 10 μM. Both the plasma (plasma sample #2) and the blood samples were incubated with the drug for 15 minutes at 37 °C (n=3). Afterwards the blood sample was centrifuged at 3000 rpm to separate the blood cells from the plasma sample (plasma sample #1). The respective plasma concentrations were determined by MS-based analysis. In a last step, the BP ratio was calculated by dividing the drug concentration in plasma sample #2 by the drug concentration in plasma sample #1. To ensure quality of the measurement, the degree of hemolysis was determined and considered negligible for all BP experiments. In addition, a control experiment without any drug was performed in parallel to determine the hematocrit of all three samples.

References

[1] Noyes AA, Whitney WR. The rate of solution of solid substances in their own solutions. J Am Chem Soc. 1897;19:930–934.

[2] Boger E, Evans N, Chappell M, Lundqvist A, Ewing P, Wigenborg A, et al. Systems Pharmacology Approach for Prediction of Pulmonary and Systemic Pharmacokinetics and Receptor Occupancy of Inhaled Drugs. CPT Pharmacometrics Syst Pharmacol. 2016;5(4):201–10. doi:10.1002/psp4.12074.

[3] Metz JAJ, Diekmann O. The Dynamics of Physiologically Structure Populations. Berlin, Germany: Springer-Verlag; 1986.

[4] Rohrschneider M, Bhagwat S, Krampe R, Michler V, Breitkreutz J, Hochhaus G. Evaluation of the Transwell System for Characterization of Dissolution Behavior of Inhalation Drugs: Effects of Membrane and Surfactant. Mol Pharm. 2015;12(8):2618–24. doi:10.1021/acs.molpharmaceut.5b00221.

[5] Olsson B, Bondesson E, Borgström L, Edsbäcker S, Eirefelt S, Ekelund K, et al. In: Smyth HDC, Hickey AJ, editors. Pulmonary Drug Metabolism, Clearance, and Absorption. New York, NY: Springer New York; 2011. p. 21–50.

[6] Wauthoz N, Amighi K. In: Nokhodchi A, Martin G, editors. Formulation strategies for pulmonary delivery of poorly soluble drugs. Chichester: John Wiley & Sons; 2015. p. 87–122.

[7] Hoffmann G, Cracknell S, Damiano J, Macri N, S M. In: Derelanko M, Auletta C, editors. Inhalation toxicology. Boca Raton, FL: CRC Press; 2014. p. 233–302.

[8] Patton JS, Byron PR. Inhaling medicines: delivering drugs to the body through the lungs. Nat Rev Drug Discov. 2007;6(1):67–74.

[9] Usmani OS. Exploring Aerosol Absorption in Humans: Pharmacokinetics of Monodisperse Fluticasone Propionate. In: Respiratory Drug Delivery 2014. vol. 1. Davis Healthcare International Publishing , LLC; 2014. p. 155–162.

[10] Weber B, Hochhaus G. A pharmacokinetic simulation tool for inhaled corticosteroids. AAPS J. 2013;15(1):159–71. doi:10.1208/s12248-012-9420-z.

[11] Applied Research Associates. Multiple-path dosimetry model version 2.11; 2009. Available from: `https://www.ara.com/products/multiple-path-particle-dosimetry-model-mppd-v-211`.

[12] Yeh HS, Schum GM. Models of Human Lung Airways and their Application to inhaled Particle Deposition. Bull Math Biol. 1980;42:461–480.

[13] International Commission on Radiological Protection. ICRP Publication 66 Human Respiratory Tract Model for Radiological Protection. vol. 24 of Annals of the ICRP. Elsevier, New York; 1994.

[14] Tamura G, Sakae H, Fujino S. In vitro evaluation of dry powder inhaler devices of corticosteroid preparations. Allergol Int. 2012;61(1):149–54. doi:10.2332/allergolint.11-OA-0332.

[15] Smith JR, Bailey MR, Etherington G, Shutt AL, Youngman MJ. Effect of particle size on slow particle clearance from the bronchial tree. Exp Lung Res. 2008;34(6):287–312. doi:10.1080/01902140802093196.

[16] Bäckman, P and Olsson, B . Pitfalls in Understanding Local Exposure.; 2016. Available from: `https://www.researchgate.net/profile/Per_Baeckman/publication/305207639_Pitfalls_in_Understanding_Local_Exposure/links/5784a4ee08ae3f355b4bab34/Pitfalls-in-Understanding-Local-Exposure.pdf`.

[17] Usmani OS, Biddiscombe MF, Barnes PJ. Regional lung deposition and bronchodilator response as a function of β_2-agonist particle size. American Journal of Respiratory and Critical Care Medicine. 2005;172(12):1497–1504. doi:10.1164/rccm.200410-1414OC.
