# Supplementary material for: A mechanistic framework for a priori pharmacokinetic predictions of orally inhaled drugs
Source: PLoS Comput Biol. 2020 Dec 15;16(12):e1008466. doi: 10.1371/journal.pcbi.1008466 (PMC7771877; doi:10.1371/journal.pcbi.1008466)
Supplement: S1 Fig — Additional sensitivity analyses for budesonide. (PDF) [file pcbi.1008466.s002.pdf]

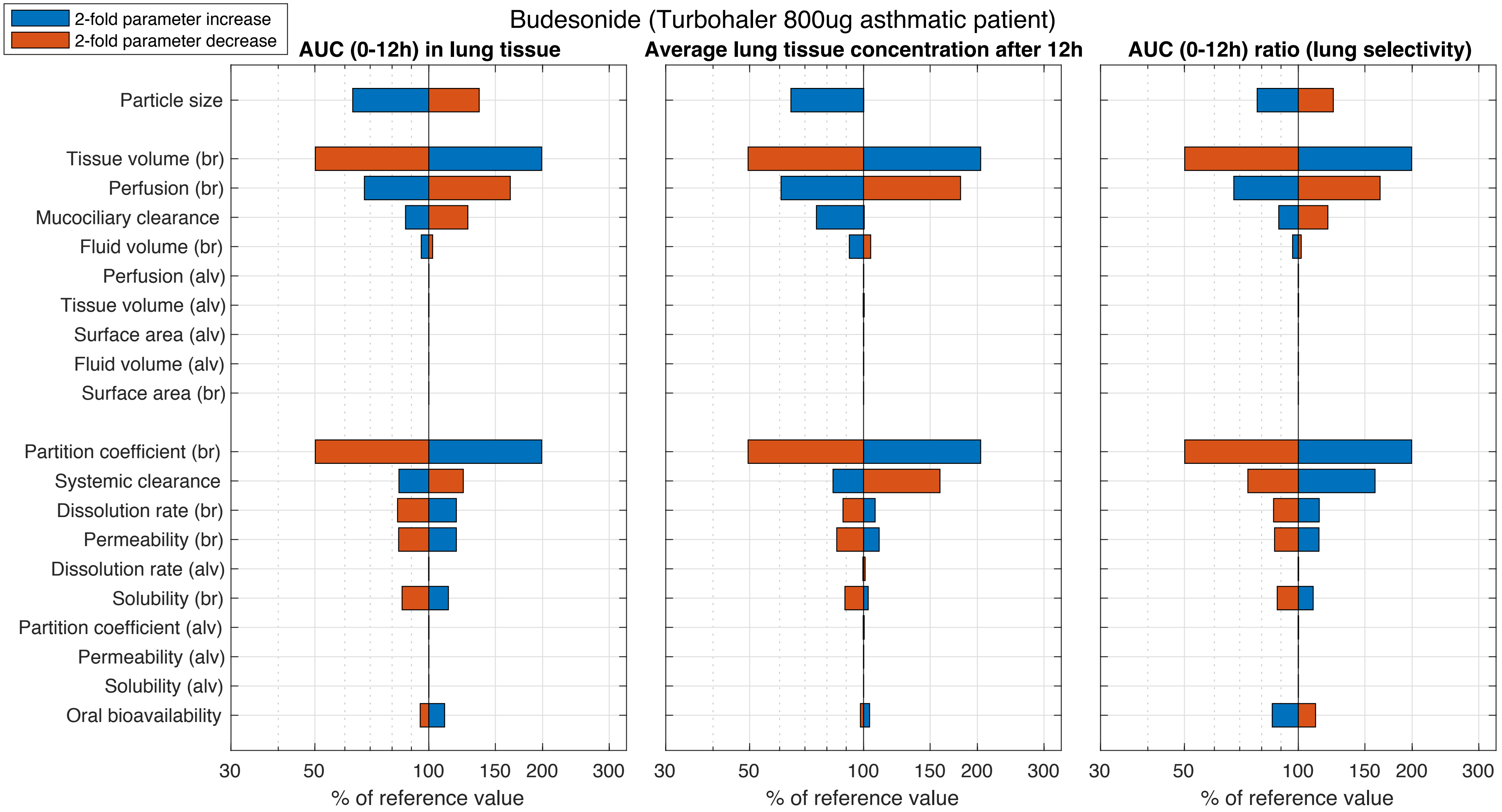

S1 Fig. Results of the performed sensitivity analysis for budesonide.

For each of three different exposure measures readouts (AUC, C12, and lung selectivity), the impact of a 2-fold increase (blue) and decrease (red) are depicted for a the formulation parameter particle size (top bar) and a set of physiological (middle bars) and drug-dependent parameters (bottom bars). The larger a bar, the stronger the impact of the varied parameter on the respective PK readout.
