# Supplementary material for: A mechanistic framework for a priori pharmacokinetic predictions of orally inhaled drugs
Source: PLoS Comput Biol. 2020 Dec 15;16(12):e1008466. doi: 10.1371/journal.pcbi.1008466 (PMC7771877; doi:10.1371/journal.pcbi.1008466)
Supplement: S2 Fig — Deposition patterns in healthy volunteers and asthmatic patients for fluticasone propionate (Diskus) and budesonide (Turbohaler). (PDF) [file pcbi.1008466.s003.pdf]

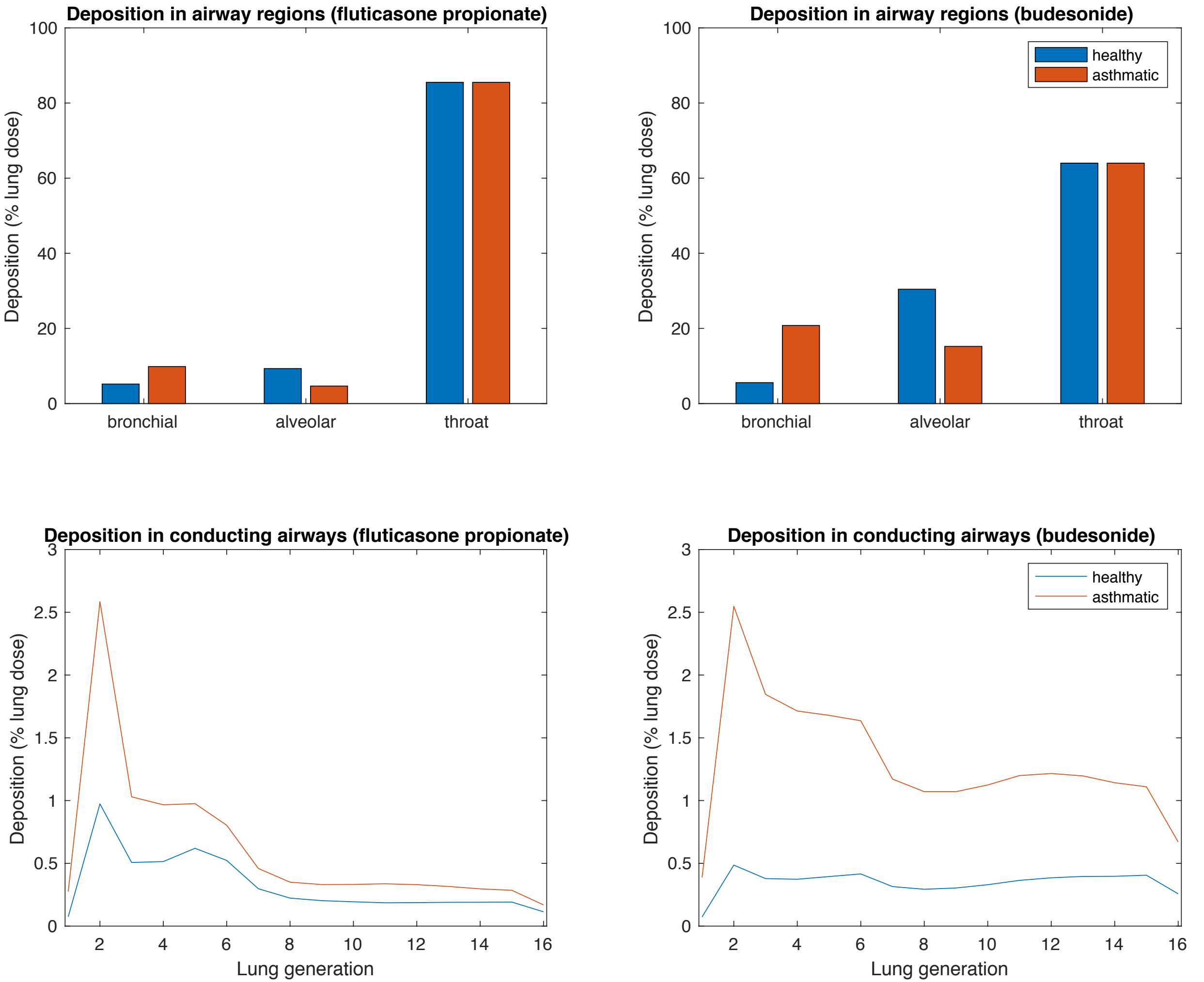

S2 Fig. Deposition patterns.

Deposition patterns in healthy volunteers and asthmatic patients for fluticasone propionate (Diskus) and budesonide (Turbohaler)
