# Supplementary material for: A mechanistic framework for a priori pharmacokinetic predictions of orally inhaled drugs
Source: PLoS Comput Biol. 2020 Dec 15;16(12):e1008466. doi: 10.1371/journal.pcbi.1008466 (PMC7771877; doi:10.1371/journal.pcbi.1008466)
Supplement: S3 Fig — Predicted time- and location-resolved lung lining fluid concentrations of fluticasone propionate (Diskus, 250 μg dose) and budesonide (Turbohaler, 800 μg dose). (PDF) [file pcbi.1008466.s004.pdf]

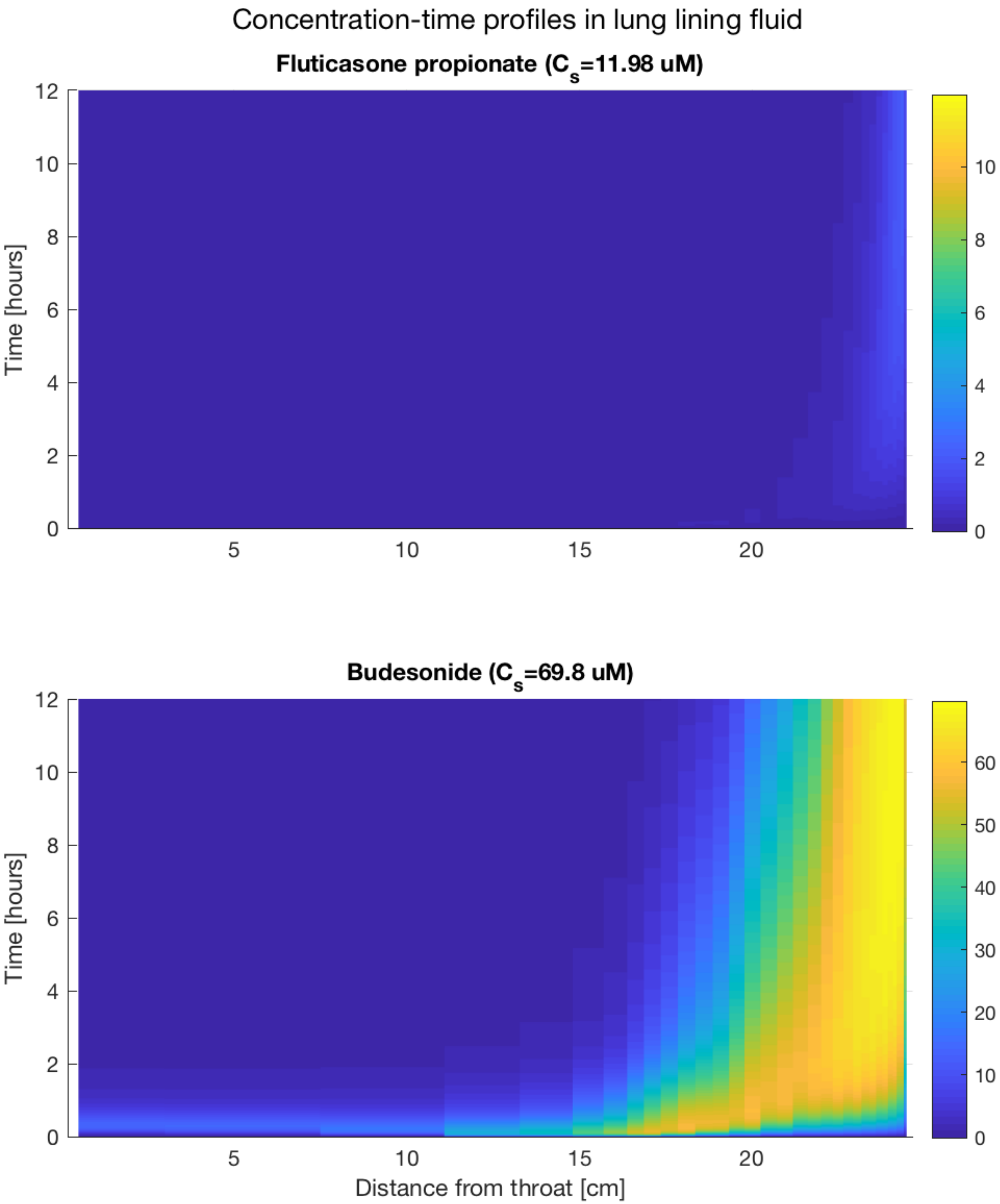

S3 Fig. Concentration in lung lining fluids.
Predicted time- and generation resolved lung lining fluid concentrations of fluticasone propionate (Diskus, 250 µg dose) and budesonide (Turbohaler, 800 µg dose).
