# Supplementary material for: A mechanistic framework for a priori pharmacokinetic predictions of orally inhaled drugs
Source: PLoS Comput Biol. 2020 Dec 15;16(12):e1008466. doi: 10.1371/journal.pcbi.1008466 (PMC7771877; doi:10.1371/journal.pcbi.1008466)
Supplement: S4 Fig — Observed vs predicted pulmonary retention of inhaled gold and polystyrene (PSL) particles (see also Fig 3 in the main text). (PDF) [file pcbi.1008466.s005.pdf]

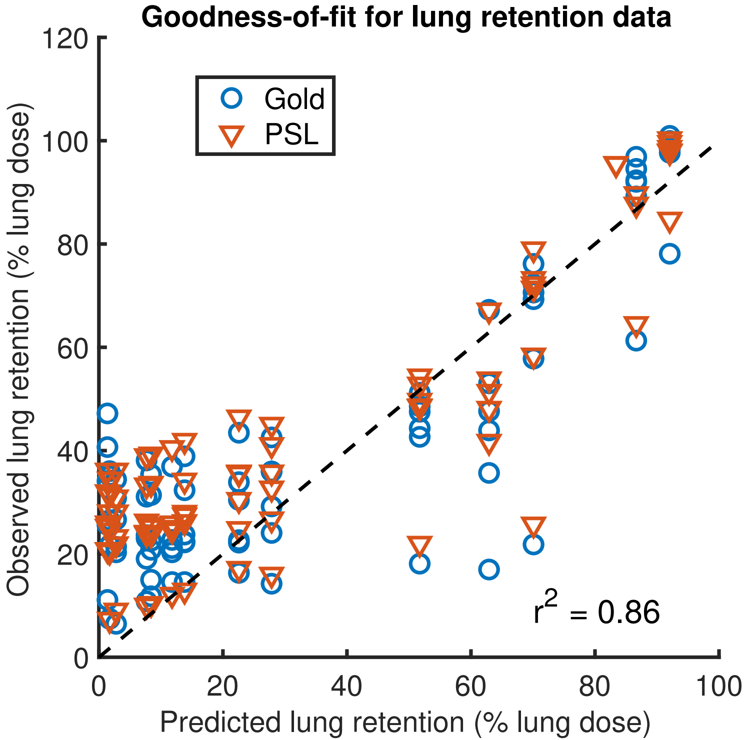

S4 Fig. Goodness-of-fit for lung retention data. Observed vs predicted pulmonary retention of inhaled gold and polystyrene (PSL) particles (see also Fig 3 in the main text).
