# Supplementary material for: A mechanistic framework for a priori pharmacokinetic predictions of orally inhaled drugs
Source: PLoS Comput Biol. 2020 Dec 15;16(12):e1008466. doi: 10.1371/journal.pcbi.1008466 (PMC7771877; doi:10.1371/journal.pcbi.1008466)
Supplement: S5 Fig — Observed vs predicted dose-normalized plasma concentrations of fluticasone propionate (left) and budesonide (right), see also Fig 4 in the main text. (PDF) [file pcbi.1008466.s006.pdf]

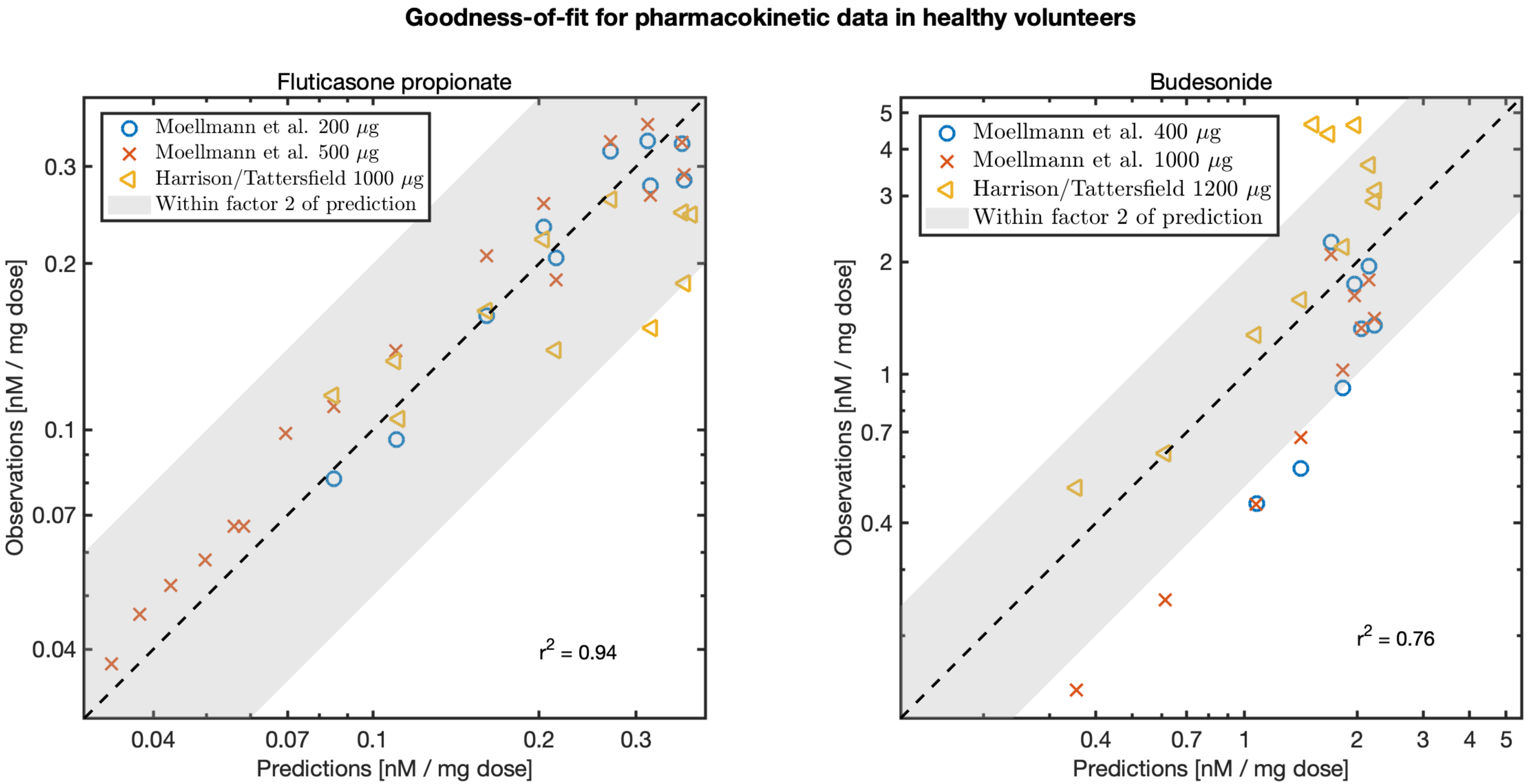

S5 Fig. Goodness-of-fit for pharmacokinetic data in healthy volunteers.
Observed vs predicted dose-normalized plasma concentrations of fluticasone propionate (left) and budesonide (right), see also Fig 4 in the main text.
