# Supplementary material for: A mechanistic framework for a priori pharmacokinetic predictions of orally inhaled drugs
Source: PLoS Comput Biol. 2020 Dec 15;16(12):e1008466. doi: 10.1371/journal.pcbi.1008466 (PMC7771877; doi:10.1371/journal.pcbi.1008466)
Supplement: S6 Fig — Solid lines: predictions for different “bolus” inhalations ranging from 100–300 mL. Data points: raw data from [26], based on a “bolus depth” of up to 135 mL. Since the bolus enters the mouth-throat at a later stage in the experimental setup by [26] compared to natural tidal breathing which is predicted with the MPPD model, the tidal volume (here “inhalation volume”) is slightly larger than the “bolus depth”, with realistic inhalation volumes of ≈ 150–200 mL. A broader range of inhalation volumes is shown to illustrate the large impact of different inhalation characteristics. (PDF) [file pcbi.1008466.s007.pdf]

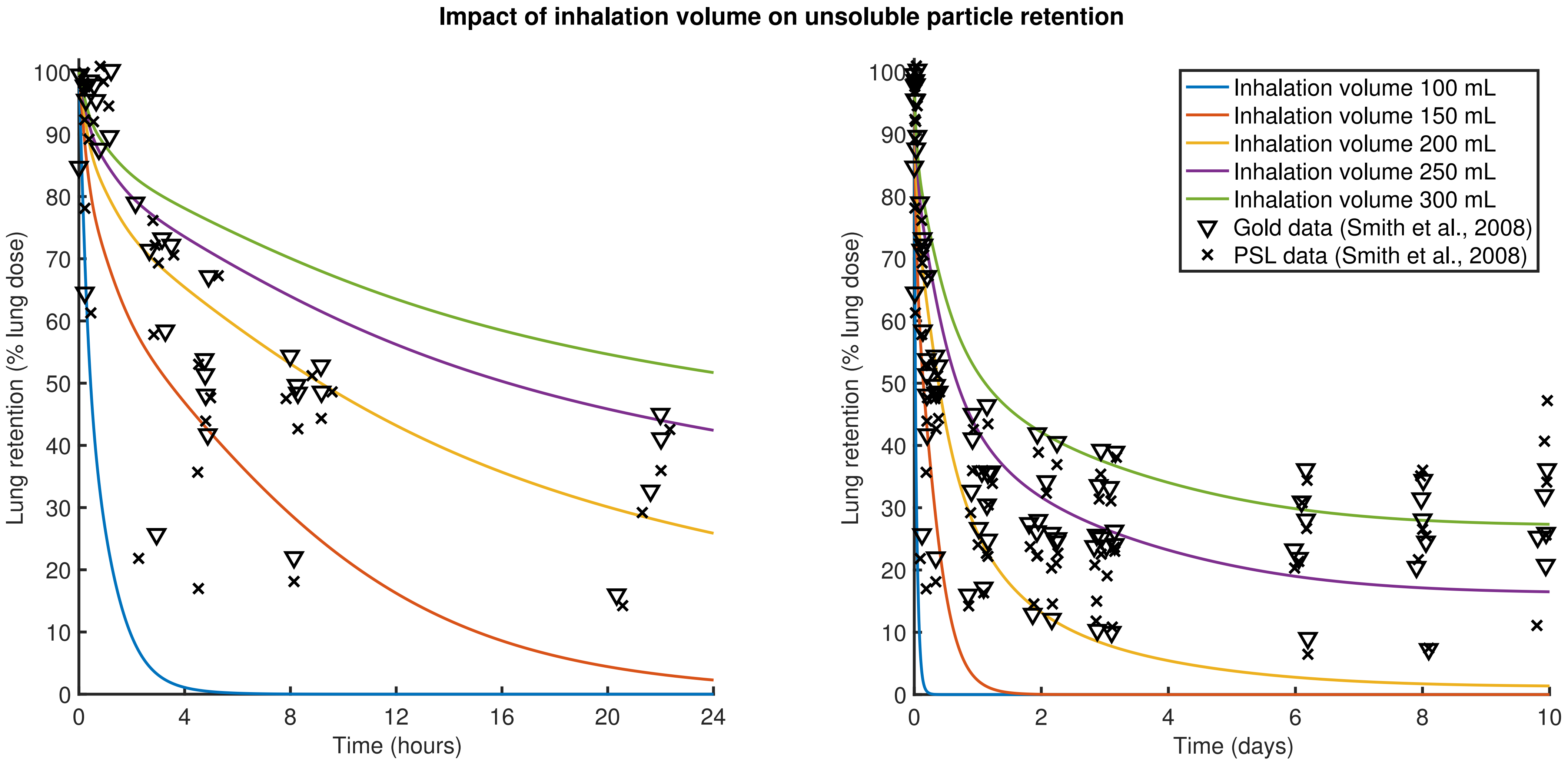

S6 Fig. Sensitivity analysis – pulmonary retention profiles for varying inhalation volumes of 5 µm particles. Solid lines: predictions for different "bolus" inhalations ranging from 100–300 mL. Data points: raw data from [1], based on a "bolus depth" of up to 135 mL. Since the bolus enters the mouth-throat at a later stage in the experimental setup by [1] compared to natural tidal breathing which is predicted with the MPPD model, the tidal volume (here "inhalation volume") is slightly larger than the "bolus depth", with realistic inhalation volumes of ≈ 150–200 mL. A broader range of inhalation volumes is shown to illustrate the large impact of different inhalation characteristics.

Reference

[1] Smith JR, Bailey MR, Etherington G, Shutt AL, Youngman MJ. Effect of particle size on slow particle clearance from the bronchial tree. Exp Lung Res. 2008;34(6):287–312. doi:10.1080/01902140802093196.
