# Supplementary material for: A mechanistic framework for a priori pharmacokinetic predictions of orally inhaled drugs
Source: PLoS Comput Biol. 2020 Dec 15;16(12):e1008466. doi: 10.1371/journal.pcbi.1008466 (PMC7771877; doi:10.1371/journal.pcbi.1008466)
Supplement: S1 Table — All identified clinical studies on fluticasone propionate and budesonide, including their study design and reported pharmacokinetic parameters. (PDF) [file pcbi.1008466.s009.pdf]

Table 1 Summary of studies for fluticasone propionate and budesonide#

Drug / substance	Study No	Description	Population	Device	Dose [µg]	Comment	References
Gold / poly-styrene	1	Insoluble mono-disperse particle	Healthy volunteers	"compressed air nebulizer"	NA	Insoluble particles to evaluate mucociliary clearance model	Smith et al. (1)
Fluticasone propionate	2	Mono-disperse particles	Asthma patients	Spinning top aerosol generator	50	Included to allow direct comparison between different inhaled particle sizes	Usmani et al. (2, 3)
	3	Sandoz Citizen Petition	Healthy volunteers	Diskus®	100, 250, 500 (in combination with 50 µg Salmeterol)	Geometric mean data of 31 individual batches	Chrystyn (5), Tarsin et al. (6), Kamin et al. (4), Sandoz Citizen Petition (7)
	4	PK study	Healthy volunteers	Diskus®	200, 500 µg (Single dose and steady state)	Study, which simultaneously provides budesonide and fluticasone propionate data	Möllmann et al. (8)
	5	PK study (IV + inhaled)	Healthy volunteers and COPD patients	pMDI + spacer	1000 µg (both iv + inhaled)		Singh et al. (9)
	6	PK study	Healthy volunteers and moderately severe asthmatics	Accuhaler®	1000 µg	Clear difference between healthies and asthmatics	Harrison and Tattersfield (10)
	7	IV PK study	Healthy volunteers	NA	250, 500, 1000 µg		Mackie et al. (11)
	8	PK study (IV + inhaled)	Healthy volunteers and mild asthmatic patients	Diskus®, pMDI	1000 µg (200 µg IV)	Publication demonstrates that systemic disposition is not different in healthies and asthmatics	Thorsson et al. (12)
	9	PK study (IV + inhaled)	Healthy volunteers	Diskhaler®, Diskus®	1000 µg (250 µg IV)		Mackie et al. (13)
	10	PK study (inhaled)	Mild-to-moderate asthmatic patients		100 / 500 µg Diskhaler, 500 µg Diskus®		Falcoz et al. (14)
	11	IV PK study	Healthy volunteers and asthmatic patients	NA	Not provided	Even though asthma and healthy data is available; no difference was investigated	Källen and Thorsson (15)
Budesonide	4	PK in healthy volunteers	Healthy volunteers	Turbohaler®	400, 1000 µg (Single dose and steady state)	Study, which simultaneously provides budesonide and fluticasone propionate data	Möllmann et al. (8)

6	PK study	Healthy volunteers and moderately severe asthmatics	Turbohaler®	1200 µg	No relevant difference between healthies and asthmatics	Harrison and Tattersfield (10)
8	PK study (IV + inhaled)	Healthy volunteers and (mild) asthmatic patients	Turbohaler®	1000 µg (200 µg IV)	Publication demonstrates that systemic disposition is not different in healthies and asthmatics	Thorsson et al. (12)
12	PK study (IV + inhaled)	Healthy volunteers	Turbohaler, pMDI	1000 µg		Thorsson et al. (16)
11	IV PK study	Healthy volunteers and asthmatic patients	NA	Not provided	Even though asthma and healthy data is available; no difference was investigated	Källen and Thorsson (15)

IV: intravenous, NA: not applicable, PK: pharmacokinetics, pMDI: pressurized metered dose inhaleder

* The deposition patterns, which were initially calculated for healthy volunteers based on the MPPD tool, were adapted based on the central/ intermediate to peripheral deposition ratios reported by Usmani et al. for asthmatic patients. FP: Fluticasone propionate, TED: total emitted dose (as % of total nominal dose)

[#] Green-shaded studies were investigated for model evaluation. Study 1 for evaluation of the deposition and mucociliary clearance model of the overall simulation framework, Study 2 to evaluate the pharmacokinetics after inhalation of different particle sizes with varying aerodynamic particle size, Study 4 to evaluate the predicted pharmacokinetics between budesonide and fluticasone propionate, and Study 6 to compare the predicted pharmacokinetics for both healthy volunteers and asthmatics. Other studies were not considered, as these did not include a comparison of one of the above listed aspects within a single study.

Table 2 PK parameters reported / calculated for the PK studies provided in Table 1

Drug / substance	Study No	Description	PK Parameters	Cmax		tmax	AUC		Comments	References
				Reported Cmax	Dose-normalized Cmax [nmol/L / mg dose]	[min]	Reported AUC	Dose-normalized AUC [nmol/L*h / mg dose]		
Gold / poly-styrene	1		Profile of particles in the lung over time	Not applicable, gold and styrene particles are mainly not being absorbed						Smith et al. (1)
FP	2	1.5 µm 50 µg	C_{max}, t_{max}, AUC_{0-12h}	425 pg/mL	17.0	8	923 pg/mL*h	36.9	Even with 100% lung dose and 100% pulmonary bioavailability, the AUC is still higher than expected	Usmani et al. (2, 3)
		3 µm 50 µg		278 pg/mL	11.1	19	891 pg/mL*h	35.6		
		6µm 50 µg		85.0 pg/mL	3.40	30	222 pg/mL*h	8.87		
	3		Dose-normalized C_{max}, t_{max}, geometric mean PK profiles							Chrystyn (5), Tarsin et al. (6), Kamin et al. (4), Sandoz Citizen Petition (7)
	4	200 µg Day 1	C_{max}, AUC, mean PK profiles	0.037 ng/mL	0.370	90	0.22 ng/mL*h	2.20		Möllmann et al. (8)
		200 µg Day 5		0.058 ng/mL	0.579	30	0.30 ng/mL*h	3.00		
		500 µg Day 1		0.094 ng/mL	0.376	90	0.79 ng/mL*h	3.16		
		500 µg Day 5		0.156 ng/mL	0.623	90	0.94 ng/mL*h	3.76		
	5	1000 µg Healthy	C_{max}, AUC, mean PK profiles	421 pg/mL	0.841	45	2996 pg/mL*h	5.99	FP administered with pMDI	Singh et al. (9)
		1000 µg COPD		235 pg/mL	0.469	45	1961 pg/mL*h	3.92	44% decreased Cmax and 35% decreased AUC compared to healthies	
	6	1000 µg Healthy	Cmax, AUC, mean PK profiles	130 pg/mL	0.260	60-120	712 pg/mL*h	1.42		Harrison and Tattersfield (10)

		1000 µg Asthmatic		78 pg/mL	0.156	60-120	404 pg/mL*h	0.807	40% decreased Cmax and 43% decreased AUC compared to healthies	
	8	1000 µg Healthy	C_{max}, AUC, mean PK profiles	0.5 nmol/L	0.5	100	3.5 nmol/L*h	3.5	Complete PK profiles only provided as a summary of healthies and mild asthmatics<br><br>Low time points considered as intention to treat (log scale jitter data points?)	Thorsson et al. (12)
		1000 µg Asthmatic		0.4 nmol/L	0.4	79	3.0 nmol/L*h	3.0	20% decreased Cmax and 14% decreased AUC compared to healthies	
	9	1000 µg Diskus	C_{max}, tmax, AUC, mean PK profiles	0.339 µg/L	0.677	25	2.49 µg/L*h	4.97		Mackie et al. (13)
	10	500 µg Diskus Asthmatics	C_{max}, tmax, AUC, mean PK profile at steady state	0.092 µg/L	0.368	40	0.474 µg/L*h	1.89	TBD	Falcoz et al. (14)
Budesonide	4	500 µg Day 1	C_{max}, AUC, mean PK profiles	0.45 ng/mL	2.09	10	0.99 ng/mL*h	4.60		Möllmann et al. (8)
		500 µg Day 5		0.48 ng/mL	2.23	30	1.22 ng/mL*h	5.67		
		1000 µg Day 1		0.90 ng/mL	2.09	10	2.53 ng/mL*h	5.88		
		1000 µg Day 5		1.10 ng/mL	2.55	20	2.98 ng/mL*h	6.92		
	6	Healthy 1200 µg	Cmax, AUC, mean PK profiles	2432 pg/mL	4.70	NA	6467 pg/mL*h	12.5	Dose-normalized AUC and Cmax for healthies ~2 fold higher than reported in Möllmann et al. (at a comparable dose)	Harrison and Tattersfield (10)
		Asthmatic 1200 µg		2276 pg/mL	4.40	NA (first time point already t_{max})	7293 pg/mL*h	14.1		
	8	1000 µg Healthy	C_{max}, AUC, mean PK profiles	3.8 nmol/L	3.8	17	10.5 nmol/L*h	10.5		Thorsson et al. (12)
		1000 µg Asthmatic		4.3 nmol/L	4.3	15	12.9 nmol/L*h	12.9		
	12	1000 µg Healthy	C_{max}, AUC, mean PK profiles	NA	NA	NA	3.5 nmol/L*h	3.5	TBD	Thorsson et al. (16)

References

1. Smith JR, Bailey MR, Etherington G, Shutt AL, Youngman MJ. Effect of particle size on slow particle clearance from the bronchial tree. Exp Lung Res. 2008;34(6):287-312.
2. Usmani OS, editor Exploring Aerosol Absorption in Humans: Pharmacokinetics of Monodisperse Fluticasone Propionate. Respiratory Drug Delivery 2014; 2014; Fajardo, Puerto Rico: Davis Healthcare International Publishing , LLC; 2014.
3. Usmani OS, Biddiscombe MF, Barnes PJ. Regional lung deposition and bronchodilator response as a function of β2-agonist particle size. American Journal of Respiratory and Critical Care Medicine. 2005;172(12):1497-504.
4. Kamin WE, Genz T, Roeder S, Scheuch G, Trammer T, Juenemann R, et al. Mass output and particle size distribution of glucocorticosteroids emitted from different inhalation devices depending on various inspiratory parameters. Journal of aerosol medicine : the official journal of the International Society for Aerosols in Medicine. 2002;15(1):65-73.
5. Chrystyn H. The Diskus: a review of its position among dry powder inhaler devices. Int J Clin Pract. 2007;61(6):1022-36.
6. Tarsin WY, Pearson SB, Assi KH, Chrystyn H. Emitted dose estimates from Seretide Diskus and Symbicort Turbuhaler following inhalation by severe asthmatics. International journal of pharmaceutics. 2006;316(1-2):131-7.
7. A. Maffia G. Sandoz Citizen Petition 2016 [Available from: https://s3.amazonaws.com/assets.fiercemarkets.net/public/005-LifeSciences/SandozCitizenPetition.pdf.
8. Möllmann H, Wagner M, Krishnaswami S, Dimova H, Tang Y, Falcoz C, et al. Single-dose and steady-state pharmacokinetic and pharmacodynamic evaluation of therapeutically clinically equivalent doses of inhaled fluticasone propionate and budesonide, given as diskus® or turbohaler® dry-powder inhalers to healthy subjects. Journal of clinical pharmacology. 2001;41(12):1329-38.
9. Singh SD, Whale C, Houghton N, Daley-Yates P, Woodcock AA. Pharmacokinetic and systemic effects of inhaled fluticasone propionate in chronic obstructive pulmonary disease. British journal of clinical pharmacology. 2003;55:375-81.
10. Harrison TW, Tattersfield AE. Plasma concentrations of fluticasone propionate and budesonide following inhalation from dry powder inhalers by healthy and asthmatic subjects. Thorax. 2003;58(3):258-60.
11. Mackie AE, Ventresca GP, Fuller RW, Bye A. Pharmacokinetics of intravenous fluticasone propionate in healthy subjects. British journal of clinical pharmacology. 1996;41(6):539-42.
12. Thorsson L, Edsbacker S, Källén A, Löfdahl C-G. Pharmacokinetics and systemic activity of fluticasone via Diskus(R) and pMDI, and of budesonide via Turbohaler. British journal of clinical pharmacology. 2001;52:529-38.
13. Mackie AE, McDowall JE, Falcoz C, Ventresca P, Bye A, Daley-Yates PT. Pharmacokinetics of fluticasone propionate inhaled via the Diskhaler and Diskus powder devices in healthy volunteers. Clinical pharmacokinetics. 2000;39 Suppl 1:23-30.
14. Falcoz C, Horton J, Mackie AE, Harding SM, Daley-Yates PT. Pharmacokinetics of fluticasone propionate inhaled via the Diskhaler and Diskus powder devices in patients with mild-to-moderate asthma. Clinical pharmacokinetics. 2000;39 Suppl 1:31-7.
15. Kallen A, Thorsson L. Drug disposition analysis: a comparison between budesonide and fluticasone. Journal of pharmacokinetics and pharmacodynamics. 2003;30(4):239-56.

16. Thorsson L, Edsbacker S, Conradson TB. Lung deposition of budesonide from Turbuhaler® is twice that from a pressurized metered-dose inhaler P-MDI. European Respiratory Journal. 1994;7(10):1839-44.
